# Relevance of Fluorinated Ligands to the Design of Metallodrugs for Their Potential Use in Cancer Treatment

**DOI:** 10.3390/pharmaceutics14020402

**Published:** 2022-02-11

**Authors:** José C. Páez-Franco, Miriam R. Zermeño-Ortega, Carmen Myriam de la O-Contreras, Daniel Canseco-González, Jesus R. Parra-Unda, Alcives Avila-Sorrosa, Raúl G. Enríquez, Juan M. Germán-Acacio, David Morales-Morales

**Affiliations:** 1 Red de Apoyo a la Investigación, Coordinación de la Investigación Científica-UNAM, Instituto Nacional de Ciencias Médicas y Nutrición SZ, Ciudad de Mexico CP 14000, Mexico; paez@cic.unam.mx; 2 Facultad de Ciencias Químicas, Universidad Autónoma de Chihuahua (UACH), Circuito Universitario S/N, Chihuahua CP 31125, Chihuahua, Mexico; mrzermeno@uach.mx (M.R.Z.-O.); cdelao@uach.mx (C.M.d.l.O.-C.); 3 CONACYT-Laboratorio Nacional de Investigación y Servicio Agroalimentario y Forestal, Universidad Autónoma Chapingo, Texcoco de Mora CP 56230, Mexico; jdcanseco@conacyt.mx; 4 School of Chemical and Biological Sciences, Autonomous University of Sinaloa (Ciudad Universitaria), Culiacan CP 80010, Sinaloa, Mexico; ricardoparraund@uas.edu.mx; 5 Departamento de Química Orgánica, Instituto Politécnico Nacional, Escuela Nacional de Ciencias Biológicas, Carpio y Plan de Ayala S/N, Colonia Santo Tomás, Ciudad de Mexico CP 11340, Mexico; aavilas@ipn.mx; 6 Instituto de Química, Universidad Nacional Autónoma de México, Circuito Exterior, Ciudad Universitaria, Ciudad de Mexico CP 04510, Mexico; habib@unam.mx

**Keywords:** organometallics, coordination complexes, metallodrugs, fluorinated ligands, anticancer activity, cancer, cytotoxicity

## Abstract

Fluorination of pharmaceutical agents has afforded crucial modifications to their pharmacological profiles, leading to important advances in medicinal chemistry. On the other hand, metallodrugs are considered to be valuable candidates in the treatment of several diseases, albeit with the caveat that they may exhibit pharmacological disadvantages, such as poor water solubility, low bioavailability and short circulating time. To surmount these limitations, two approaches have been developed: one based on the design of novel metallodrug-delivering carriers and the other based on optimizing the structure of the ligands bound to the metal center. In this context, fluorination of the ligands may bring beneficial changes (physicochemical and biological) that can help to elude the aforementioned drawbacks. Thus, in this review, we discuss the use of fluorinated ligands in the design of metallodrugs that may exhibit potential anticancer activity.

## 1. Introduction

Many metallodrugs have been used throughout human history; current methods and analytical techniques have helped to identify and elucidate therapeutic targets and mechanisms of action [1]. The therapeutic applicability of the first metallodrugs was discovered in a serendipitous manner. For instance, Salvarsan (also known as arsphenamine, or Ehrlich 606) was developed by Ehrlich and Bertheim in 1910 [2]. It is a mixture of 3-amino-4-hydroxyphenyl-As^III^ compounds. Salvarsan was marketed by Hoechst as a therapeutic agent for the treatment of syphilis, and later regarded as a chemotherapeutic agent. Additionally, the revolutionary discovery of the antitumoral drug cisplatin by B. Rosenberg in the 1960s had a profound impact on cancer chemotherapy [3]. Since then, three platinum-based metallodrugs have been approved for the treatment of cancer in humans: cisplatin, carboplatin, and oxaliplatin [4].

In addition, several other metallodrugs have been approved in the US and Europe [1] encompassing diverse metals and metalloids with different therapeutic applications, as anticancer agents, antimicrobials, antivirals, antidiabetics and anti-HIV agents, among others [1,5,6,7].

**Figure 1:**
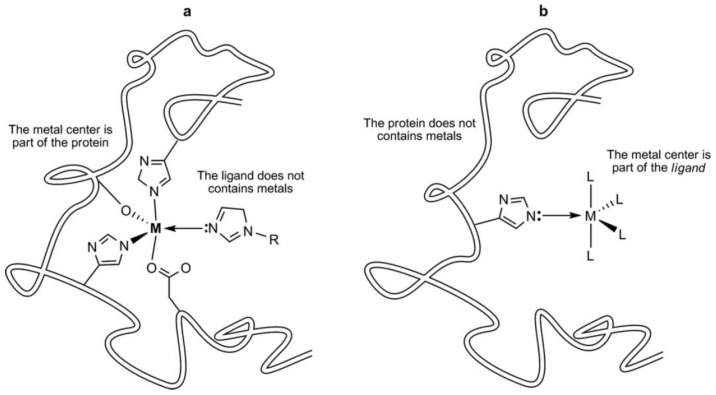
The two possibilities of interaction between a metal center and a ligand: (**a**) a coordination bond between the metal center of a metalloprotein and an organic ligand; (**b**) coordination bond between a metal-containing ligand and a protein side chain. Reproduced with permission from [14], Wiley, 2018.

Within the realm of metallodrug design, the ligands involved play a pivotal role [8]. In this regard, the exploration of metal–ligand interactions for the design of new drugs is an important issue. Thus, the understanding at an atomic/molecular level of the metal–ligand interactions is a very important tool for the exploitation of any potential medical use. Further, the metallic center in metallodrugs also plays a fundamental role because, (1) the metal confers geometries not possible with other elements (square planar, square pyramidal, octahedral, etc.), allowing regio- and stereospecific structures; (2) the metal center can provide catalytic, redox (various oxidation states) and photophysical properties, among others. Another detail to consider is that the ligands bound to the metal center confer flexibility and dynamics and globally define the electronic structure of the metallodrug complex they form. Noteworthy is the fact that in metallodrugs, the ligands can be displaced or dissociated into one or more labile ligands, suffering chelate ring-opening, or the ligand can just modulate the redox properties of the metal [8,9]. Most of these properties are hard to find in organic drugs. Another very important function of ligands in metallodrugs is the fact that they can selectively activate the metallodrugs at the target site by the effect of an external stimulus such as light, radiation, sound or heat [1,10,11]. Thus, the metal center in metallodrug serves as a structural element that helps organize and present the ligands at the binding site of the target proteins (Figure 1) [12]. For instance, the success of the DNA–cisplatin recognition process depends on several conditions: cisplatin transport, its cellular uptake, biodistribution and toxicity profile [13]. Thus, before the DNA–cisplatin interaction occurs, another series of protein–cisplatin interactions must be established. In this sense, molecular docking studies facilitate the rational design of pharmaceutically relevant metallodrugs [14].

**Figure 2:**
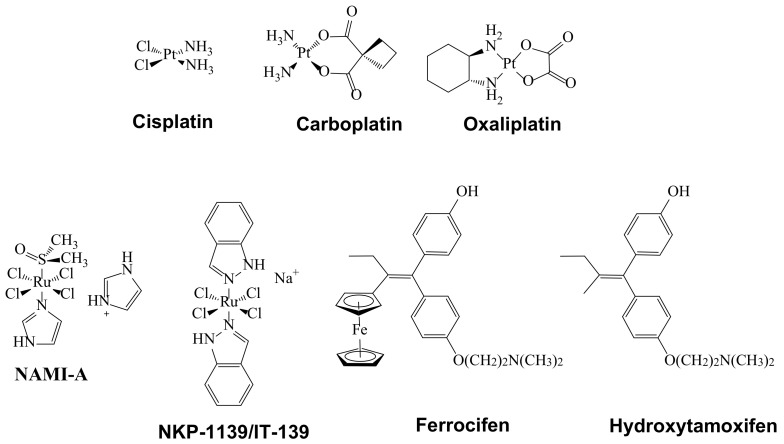
Diverse metallodrugs with anticancer properties.

Various metallodrug candidates in clinical trial phases have shown several limitations, mainly due to poor pharmacokinetic properties [15]. For example, since its discovery, cisplatin has been one of the best-selling antitumor agents in the world [16]. However, it exhibits undesirable side effects, such as nephrotoxicity, ototoxicity, peripheral neurotoxicity and resistance problems. It is because of this that other Pt-based metallodrugs have appeared (i.e., carboplatin and oxaliplatin) and gained international marketing approval (Figure 2). Other Pt-based metallodrugs such as nedaplatin, lobaplatin and heptaplatin have only been approved in a few countries [16]. Moreover, metallodrugs such as Satraplatin, Picoplatin, Lipoplatin^TM^ and ProLindac^TM^ were until 2014 still at some point in their clinical trial phases [16]. In addition, with the aim of eliminating the above mentioned side effects, still other non Pt-based metallodrugs including Ruthenium derivatives have been developed: for instance, trans-[tetrachloro(DMSO)(imidazole)ruthenate(III)] (NAMI-A), indazolium trans-[tetrachlorobis(1*H*-indazole)ruthenate(III)] (KP1019) and its sodium salt analogue NKP-1339. These metallodrugs have demonstrated high selectivity for solid tumor metastases and low toxicity at pharmacologically active doses. Noteworthy is the fact that NKP-1139/IT-139 exhibits excellent water solubility and because of this, it is currently in clinical phase studies (Figure 2) [17,18].

Ferrocifen is a metallodrug that exhibits excellent permeation capacity, by being a more lipophilic species compared to hydroxytamoxifen (Figure 2) [19,20]. Hence, Ferrocifen can easily cross cell membranes, showing better cytotoxic effects than Hydroxytamoxifen, a selective estrogen receptor modulator widely used in the treatment of hormone-dependent breast cancer, but that unfortunately often leads to resistance after long-term administration. In addition to Ferrocifen, several other metallodrugs regarded as chemotherapy agents containing other metal centers (Au, Fe, Ag, Ga, Rh, and Ti) have been reported to show potential anticancer properties and have entered clinical trials [16].

As can be seen, important progress in the preparation of diverse new metal-based anticancer drugs has been made in recent years. However, most of these molecular species exhibit different pharmacological drawbacks (i.e., poor water solubility, low bioavailability and short circulating time) [21] that have led to the design of novel metallodrug-delivering vehicles [22,23,24,25,26] and the structural optimization of the ligand(s) in order to overcome these hurdles [21].

Thus, in this review we were interested in structural optimization approaches, specifically those dealing with the fluorination of the ligands bound to the metal center. As is commonly known, incorporation of fluorine atom(s) on the ligands can produce important physicochemical and biological improvements [27]. Fluorine is the second smallest atom and the most electronegative element in the periodic table, providing different chemical properties compared with the hydrogen atom [28]. Fluorine chemistry has had an important role in medicinal chemistry, mainly in drug design [29,30,31,32]. Due to its inductive effect, fluorine can reduce the basicity and enhance the acidity of numerous compounds, thus improving the membrane penetration of fluorinated compounds due to increased lipophilicity and by reducing the number of charges present in the molecules [33]. These properties allow modulation of the solubility, permeability, and protein binding of the compounds. In addition, fluorine atoms can participate in a great variety of intermolecular interactions that may enhance their binding affinity towards a given target protein [28]. Further, the formation of C-F bonds within the fluorinated compounds provides thermal and chemical robustness to avoid degradation. However, a caveat found with the fluorination of pharmaceutical agents is their decreased solubility in water, hence making relevant the proper modulation of the number of fluorine atoms incorporated in their structures [27].

Thus, in this paper we review different articles related to metallodrugs bearing fluorinated ligands with interesting anticancer activities. Transition metal complexes including fluorinated-NHC (N-heterocyclic carbenes) with anticancer properties are outside the scope of this paper, as they have been recently reviewed [34].

## 2. Cadmium Metallodrugs

In recent years, quantum dots (QDs) have gained relevance in biomedical applications mainly as sensing and imaging active agents [35,36]. QDs exhibit various advantages such as fluorescence abilities, optical properties (high quantum yields), large extinction coefficients, photostability and broad absorption spectra coupled to narrow size-tunable photoluminescent emission spectra [35]. However, deficient cellular internalization is one of the disadvantages of these structures [37]. To overcome this limitation, high doses of QDs are most often used. In this way, it has been pointed out that the interaction between the QDs and the cell membrane is a key factor [37]. Fluorination of the capped ligands appears to be an effective way to improve the internalization problem since the polarity of the QDs is modified [38]. With this in mind, Argudo et al. prepared QDs containing CdSe/ZnS decorated with the fluorinated ligand HS-C_11_H_22_-(EG)_4_-O-C(CF_3_)_3_ (EG = ethyleneglycol) [37]. Through computational and experimental studies, they evaluated the internalization processes of these fluorinated QDs through a cell membrane simulated by a monolayer of the phospholipid 1,2-dipalmitoyl-sn-glycero-3-phosphocholine (DPPC). Surface pressure-molecular area isotherms indicated that fluidization of DPPC molecules was caused by direct interaction with the fluorinated QDs. Additionally, molecular dynamics simulations showed that DPPC (alkyl chains) molecules were occluded onto the coating layer of the fluorinated QDs. Cellular uptake in vitro experiments in HeLa cells exhibited that cell viability decreased to 85% after 5 h of incubation with QDs. After 24 h of incubation, the cell viability decreased to 75%. A significant cellular uptake was proved by confocal fluorescence microscopy. Thus, authors described that fluorination in the ligands helped to modify the physicochemical and biological properties, resulting in the efficient internalization of the cell trough the cell membrane.

## 3. Cobalt Metallodrugs

**Figure 3:**
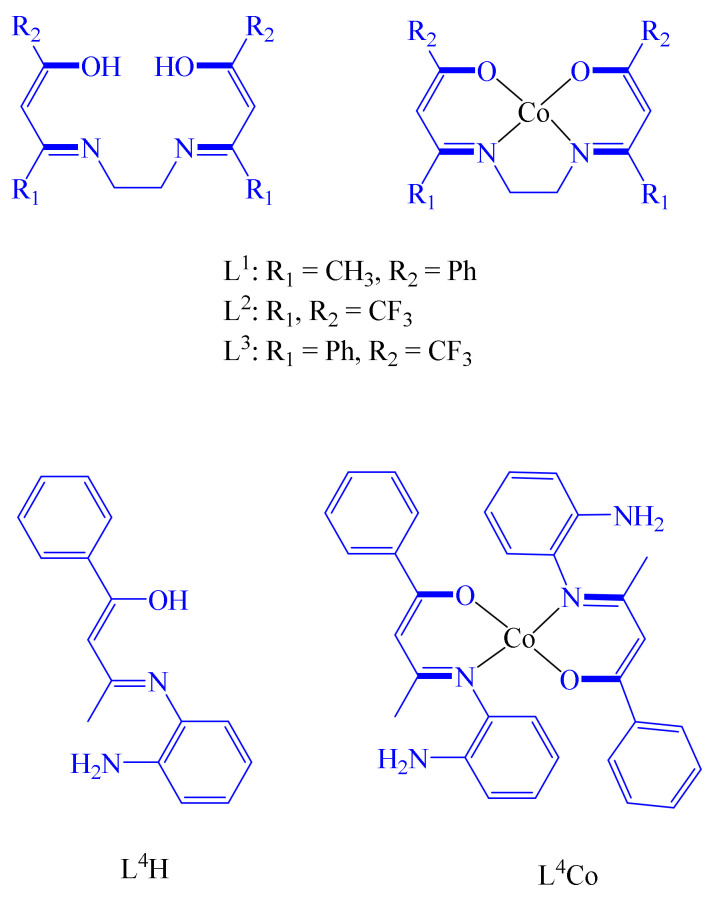
Diverse Co(II)-based metallodrugs with anticancer properties.

Gurley et al. reported that there are only a few studies describing Co-based metallodrugs with potential therapeutic applications [39]. For instance, Ott et al. informed the preparation and antitumor efficacy of [2-acetoxy-(2-propynyl)benzoate] hexacarbonyldicobalt [40]. Although the mechanism of action of this specie is unclear, it is suggested that it occurs via DNA–metal complex interaction [41]. Gurley et al. indicated their interest in using fluorinated ligands directly bound to a Co center. This is because fluorination can cause significant changes (physicochemical and biological) in the metallodrug. Hence, they designed at least two different types of ligands: (a) tetradentate bis(β-ketoaminato) (L^1^–L^3^) and (b) hydroxyimine (L^4^), Figure 3. It is noteworthy that L^2^ and L^3^ are fluorinated ligands. These ligands were coordinated to Co(II) metal centers to produce the corresponding complexes L^1^Co–L^4^Co.

In this study, authors determined the half-maximal effective concentration (EC_50_) values on six different human tumor cell lines, i.e., PC-3 (prostate), VCaP (prostate), THP-1 (acute monocytic leukemia), U-373 MG (astrocytoma), HepG2 (liver carcinoma) and SH-SY5Y (neuroblastoma), using both the free ligands and the corresponding metallodrugs. In addition, special emphasis was placed on observing the effect of the fluorinated ligands.

According to the EC_50_ values, Co-based metallodrugs showed better cytotoxic activity than the free ligands. Moreover, among the cobalt complexes, L^2^Co exhibited the best activity and L^4^Co the lowest. Particularly interesting is the fact that L^2^Co showed a notable effect on PC3 cells (94% maximal inhibition and an EC_50_ of 2.5 μM). In general, metallodrugs L^1^Co–L^4^Co exhibited selectivity against two prostate cancer cell lines and monocytic leukemia cells (THP-1). Authors also mentioned that in compound L^2^Co, the presence of the four CF_3_ groups helped to potentiate its cytotoxicity towards both human cancer cell lines, while the other two complexes, L^1^Co and L^3^Co, exhibited lower cytotoxicity values, with L^4^Co being the least effective. According to the authors, previous studies have revealed that tetrahedral Co(II) complexes had very low cytotoxicity towards a range of human cancer cell lines compared with the corresponding square planar species. In this case, compound L^4^Co (tetrahedral geometry) showed modest activity compared with derivatives L^1^Co–L^3^Co (square planar). Furthermore, experiments aimed to determine the possible mechanism of action of complexes L^1^Co and L^2^Co showed the latter to induce the activation of caspase-3 in PC-3 cells at concentrations of either 5 or 10 μM. Experimental findings regarding L^1^Co showed this effect to be weaker because the statistical data were significatively poor. The significant caspase-3 activation by L^2^Co indicates a mechanism of action similar to that of cisplatin, suggesting that cell death is induced by apoptosis [42]. In addition, other experiments showed that the cytotoxic activity of complexes L^1^Co–L^3^Co against PC-3 cells is dependent on the activation of MAP kinases and c-Jun NH_2_-terminal kinase (JNK). The activation of these proteins thus implies, once again, a mechanism by apoptosis similar to that observed with cisplatin [41,43]. Thus, overall, this work shows that the metallodrug L^2^Co (Figure 3) exhibits important cytotoxic activity towards prostate cancer and leukemia cells and that this enhanced activity is probably caused due to the fluorination effect of the ligand.

More recently, in 2019, King et al. described the preparation of a series of new Co(III)-Schiff base complexes [44]. The authors were interested in Co(III) complexes due to the significant effect of this kinetically inert ion on ligand substitution reactions, causing them to proceed more slowly compared to other metal ions such as, Co (II), which produces more labile species. Hence, special emphasis has been placed on the development of Co(III) prodrugs that can be reduced in biological environments to the more labile Co(II) form, which can more easily release the ligand at the target site [45]. The slow ligand exchange kinetics in octahedral Co(III) complexes has an important contribution in the substitution rate of the ligands at the enzymatic site, which can dramatically affect their activity [44].

**Figure 4:**
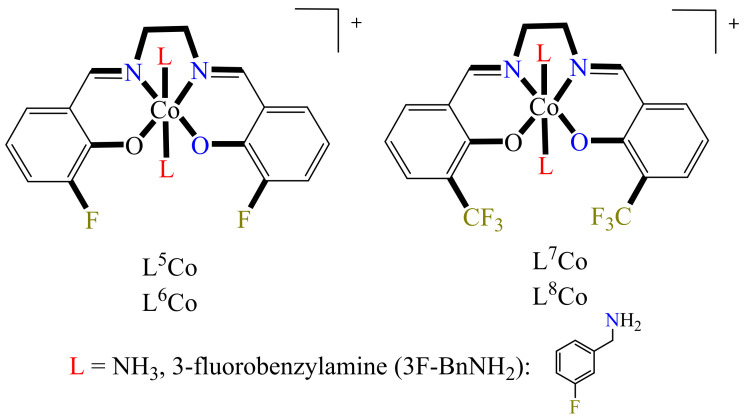
Diverse Co(III)-based metallodrugs with anticancer properties.

In this regard, the authors prepared a series of four Co(III) complexes, holding at the axial position molecules of NH_3_ or 3-fluorobenzylamine (3F-BnNH_2_) (Figure 4) (L^5^Co–L^8^Co). At the equatorial position, the metal center is coordinated to the ligands 3-fluorosalicylaldehyde ethylenediamine (3F-salen) or trifluoroacetylacetone ethylenediamine (tfacen). Here, the interest in the use of fluorinated ligands is mainly due to the following: (1) the study of the speciation in solution can be performed via ^19^F NMR, (2) the equatorial fluorinated ligands confer slow ligand substitution on the Co(III) center, and (3) these fluorinated compounds can be analyzed via ^19^F magnetic resonance imaging (MRI) or by ^18^F positron emission tomography.

**Figure 5:**
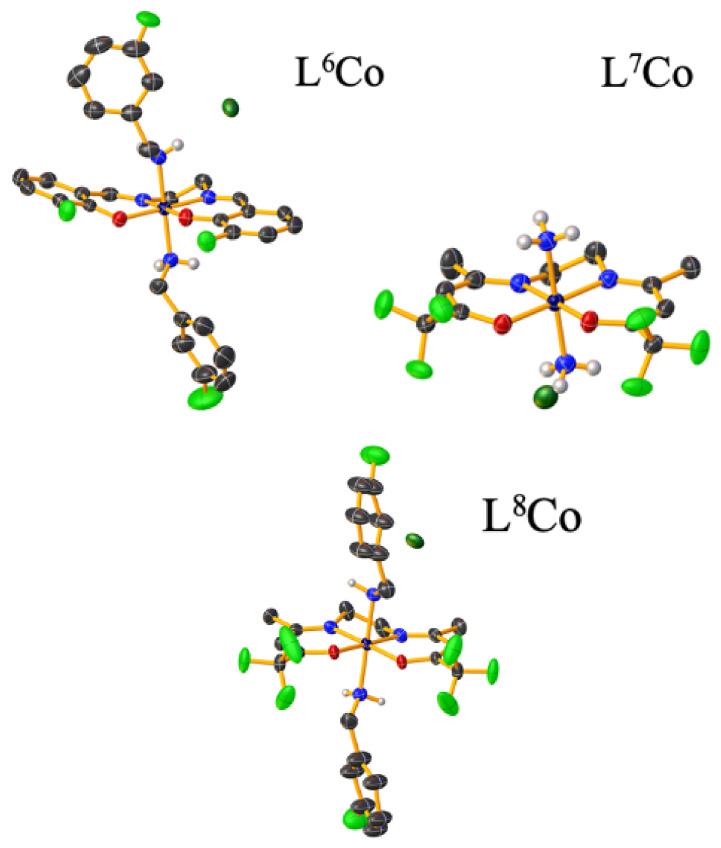
Crystal structures of complexes L^6^Co–L^8^Co. Some hydrogen atoms of the corresponding anion were omitted for clarity. CSD database identifier: 1864183–1864185.

Luckily the three Co(III) complexes were crystallized: (1) [Co(3F-salen)(3F-BnNH_2_)_2_]Cl·DMF (L^6^Co), (2) [Co(tfacen)(NH_3_)_2_]Cl·MeOH (L^7^Co), (3) [Co(tfacen)(3F-BnNH_2_)_2_]Cl·Et_2_O (L^8^Co) (Figure 5). Further, their single-crystal X-ray diffraction (SCXRD) analyses confirmed that the complexes (L^6^Co–L^8^Co) have a distorted octahedral geometry, with the Schiff base ligands 3F-salen or tfacen occupying the equatorial positions with the NH_3_ or 3F-BnNH_2_ ligands, being coordinated axially. Further analysis by cyclic voltammetry (upon scanning cathodically) revealed that complexes L^6^Co–L^8^Co were reduced to Co(II) irreversibly due to the loss of the axial ligands, while anodic scanning showed that the Co(II) species was oxidized to Co(III), presumably forming the solvent adduct [Co(Schiff base)(DMF)_2_]^+^. The peak potential of the Co(III)/Co(II) pair for the complexes bearing the tfacen ligands was approximately 100 mV more negative than that determined for the 3F-salen derivatives. However, when considering the axial ligands, the complexes having NH_3_ were 150 mV more negative than their analogues with the 3F-BnNH_2_ ligand.

**Scheme 1:**
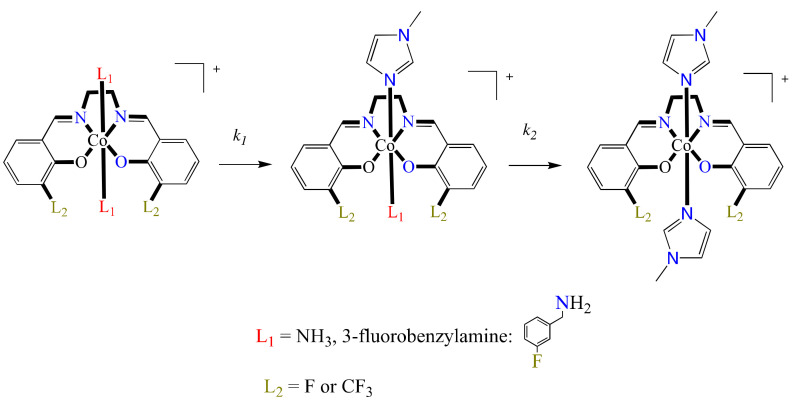
Proposed ligand exchange pathway for [Co(Schiff base)(L)_2_]^+^ complexes.

Additionally, for the same series of complexes, kinetic lability studies of the axial ligands were performed using ^19^F NMR spectroscopy and RP-HPLC. These experiments were performed in the presence of N-methylimidazole (MeIm) as a model for a protein histidine side chain (Scheme 1). The results showed no dependence on the concentration of MeIm, suggesting a zero-order reaction related to a dissociative mechanism, as the rate of the ligand substitution largely depended on the leaving axial ligand. Thus, as a ligand with greater donor ability than 3F-BnNH_2_, NH_3_ has a slower exchange rate. In addition, Eyring’s analysis of the ligand exchange reactions at different temperatures showed positive activation entropy values consistent with a dissociative mechanism. Furthermore, Co uptake and cytotoxicity experiments were also performed. In vitro cytotoxic assays were performed against A549 lung cancer cells using thiazolyl blue tetrazolium bromide MTT. IC_50_ values were determined from concentrations of 50 μM to higher than 500 μM. The results showed that complexes bearing NH_3_ ligands were less cytotoxic than those containing ligand 3F-BnNH_2_ at the axial positions. A similar situation was observed in the Co uptake experiments, with complexes containing NH_3_ ligands exhibiting smaller absorption values than the 3F-BnNH_2_-complexes. This was a consequence of NH_3_-complexes being less lipophilic and the slower ligand exchange limiting the intracellular reduction of Co(III) to Co(II), conversely to that observed on the complexes having ligand 3F-BnNH_2_. Thus, in general, NH_3_ complexes were cytotoxically inactive, while those derivatives including ligand 3F-BnNH_2_ exhibited moderate activity, hence showing that fluorination of the ligands can produce a change in the biological properties of a given complex.

## 4. Copper Metallodrugs

**Figure 6:**
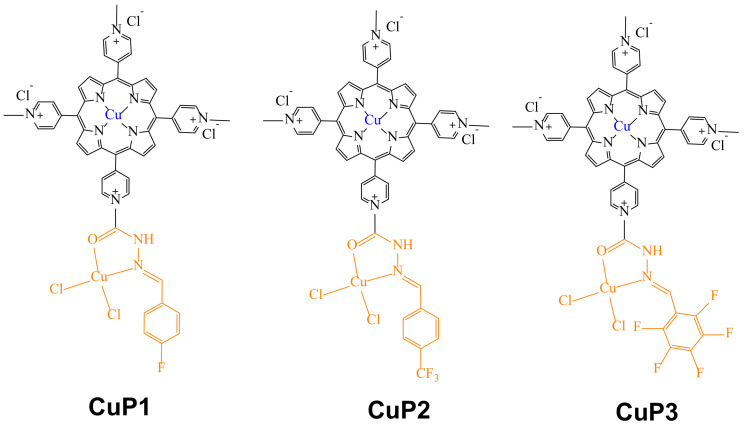
A series of acyl-hydrazone porphyrin Cu(II)-complexes with anticancer properties.

Copper is an essential trace element with fundamental function in many enzymes related to energy metabolism, mitochondrial respiration (e.g., cytochrome oxidase; Cco), antioxidation (e.g., Zn, Cu-super-oxide dismutase; SOD), collagen cross-linking (e.g., lysyl oxidase), pigmentation (e.g., tyrosinase), and cathecolamine biosynthesis (e.g., dopamine- β-monooxygenase) [46]. In part because of this, copper-based metallodrugs have attracted much attention for the treatment of tumor angiogenesis [47]. In fact, various copper-based complexes have been studied in vitro and in vivo. Studies have gathered a large volume of information indicating that the nature of the ligand plays an important role in the anticancer activity of copper complexes [48]. Hence, several ligands have been used for coordination to Cu(I/II) ions, including thiosemicarbazones, 8-hydroxylquinoline, imidazoles, pyridine N-Oxides, Schiff bases, hydrazones, and macrocyclic ligands [46,49]. However, most of these copper complexes have limited water solubility, making it difficult to find suitable medicinal applications for them. Thus, Li et al. engaged in the design of novel water-soluble acyl-hydrazone porphyrin Cu(II)-complexes (CuP1-CuP3) (Figure 6) [50], including fluorine on the acyl-hydrazone moieties and pyridinium fragments to improve their water solubility.

With the complexes on hand, UV-visible binding affinity experiments were carried out using circulating tumor DNA, with results showing that DNA-complexes have the following trend: CuP3 > CuP1 > CuP2. Large hypochromicities were found, implying that DNA-metallodrug interactions occur through intercalated binding accompanied by end-stacking. The intrinsic binding constants (K_b_) were estimated using the Stern–Volmer equation, obtaining values of K_b_ of 1.84 × 10^4^, 1.82 × 10^4^, 3.23 × 10^4^ M^−1^, respectively, indicating that complex CuP3 had the highest affinity for DNA, a finding consistent with the hypochromism trend observed above.

In addition, intercalation ethidium bromide (EB) EB-DNA experiments were performed. The fluorescence intensity of EB in aqueous solution is very weak; however, once EB-DNA intercalation is established, the fluorescence intensity is enhanced considerably, and the fluorescence intensity of EB-DNA can be reduced when another intercalating agent is competing for the DNA binding sites. Thus, in these competitive reactions of EB-DNA in the presence of the complexes of the series CuP1–CuP3, a decrease in the fluorescence intensity of the EB-DNA adduct was observed, indicating that EB was displaced, thus modifying the DNA conformation. Hence, quenching constants K_q_ were determined by the Stern–Volmer equation, finding that the K_q_ values for the DNA-complexes were 8.94 × 10^5^, 8.50 × 10^5^ and 9.18 × 10^5^ for CuP1, CuP2 and CuP3, respectively. In addition, the apparent DNA binding constants (K_app_) were also calculated. First, the K_app_ value for the adduct EB-DNA was determined to be [EtBr] = 5 μM, K_EtBr_ = 1.0 × 10^7^ M^−1^. Then the K_app_ values for the DNA-complexes were determined to be 1.54 × 10^7^ M^−1^ (CuP1), 1.33 × 10^7^ M^−1^ (CuP2) and 1.54 × 10^7^ M^−1^ (CuP3), clearly showing that in fact the adduct CuP3-DNA was the one exhibiting the highest binding affinity. This is in accordance with the results obtained from the UV-visible experiments.

Cytotoxic assays were also performed for the complexes CuP1, CuP2, and CuP3 and their corresponding free ligands L1, L2, and L3 (Figure 6). All compounds were tested by MTT assays against A549 and H-1975 lung cancer cells, HepG2 liver carcinoma cells, T47D breast cancer cells and Hs 578Bst normal breast cells. The results showed the three complexes CuP1, CuP2, and CuP3 to effectively inhibit activity in four cell lines, except for Hs 578Bst normal breast cells in which the cytotoxicity was low, whereas the free ligands exhibited low cytotoxic activity against all the cell lines even at a relatively high concentration of 200 μM, thus indicating that coordination of Cu(II) on the ligands results in enhanced cytotoxic activities. Further, based on the half-maximal inhibitory concentration (IC_50_) values, complex CuP3 exhibited good inhibitory power in all cell lines, showing the best anti-oncocyte activity and the lowest IC_50_ value after 72 h of treatment for the H-1975 lung cancer cell line. The authors credited this outstanding cytotoxic activity of complex CuP3 to the fact that it contains more fluorine atoms in its structure, thus causing an improvement in its lipophilic properties.

In addition, using the same series of complexes, cellular uptake experiments were performed towards four different malignant tumor cell lines, i.e., A549, H-1975, HepG2, and T47D. Overall, the CuP3 complex showed the best cellular uptake behavior, being particularly effective against the H-1975 tumor cell line. As noted before, this behavior was explained by the fact that complex CuP3 has the highest number of fluorine atoms in its structure, increasing its amphiphilicity and thus improving its cellular uptake. These results were further confirmed by fluorescence microscopy and cell cycle distribution analysis.

Hence, Hoechst 33342 and propidium iodide (PI) experiments allowed quantification of the population of live and dead cells in tumor H-1975 after treatment with CuP3. Cells untreated with CuP3 exhibited the blue Hoechst 33342 fluorescence (control group), and those treated with CuP3 exhibited the red PI fluorescence. These experiments demonstrated that the cytotoxic activity of CuP3 against H-1975 cells is time- and dose-dependent. So, increasing the concentration and duration of treatment with CuP3 (at 48 h) led to the highest cell mortality rate. Further, fluorescence microscopy tests showed that after being treated with CuP3 for 48 h, the nuclei of H-1975 cells were condensed or fragmented, demonstrating the apoptotic effect of the complex. With this work, the authors proved that the amphiphilicity of CuP3 was enhanced, because this compound is the most fluorinated of the series.

**Figure 7:**
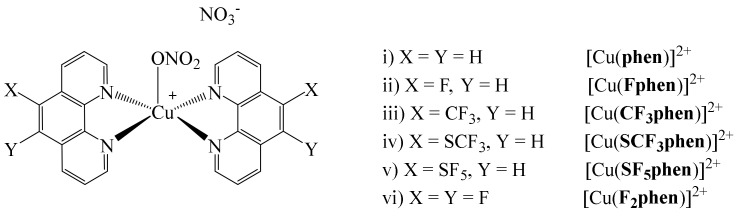
Cu(II) complexes with fluorinated phen ligands.

More recently, in 2021, Lüdtke et al. reported the preparation of a series of homoleptic Cu(II) complexes of the type [Cu(phen)_2_]^2+^ (phen = 1,10-phenanthroline) (Figure 7) [51], with 1,10-phenanthroline ligand being modified by incorporation of different fluorinated substituents.

Sigman and coworkers, over 4 decades ago proved the nucleolytic activity of complex [Cu(phen)_2_]^2+^ against DNA in the presence of O_2_ and 3-mercaptopropionic acid [52]. The thiol is used as reducing agent to produce Cu(I) complexes that may yield reactive oxygen species (ROS) able to cause damage to DNA. According to the authors, further experiments using similar complexes of the type [CuL_2_]^2+^ (L = phen derivatives: quinoxaline, phenazine, etc.) for oxidative cleavage of DNA made necessary the use of reducing agents to produce ROS. In this regard, the inclusion of fluorinated substituents on the phen ligands may help to tune the redox properties of the complexes [Cu(phen)_2_]^2+^; the fluorinated phen ligands then may act as chemical nucleases.

The determination of the half-wave potentials E_1/2_ of the Cu(II) complexes showed that the compounds with the highest electron-withdrawing abilities better stabilized the Cu(I) species, achieving a nice correlation between the determined half-wave potential values and the electron-withdrawing abilities of the complexes as follows: H < F < CF_3_ ≈ SCF_3_ < SF_5_. Then, these complexes were tested on the cleavage towards supercoiled plasmid DNA to evaluate their nucleolytic activity. These experiments were performed in the presence and absence of a reducing agent (ascorbic acid). It was observed that in the presence of ascorbic acid, the efficiency on the cleavage was: [Cu(phen)_2_]^2+^ > [Cu(Fphen)_2_]^2+^ > [Cu(F_2_phen)_2_]^2+^ > [Cu(CF_3_phen)_2_]^2+^ > [Cu(SCF_3_phen)_2_]^2+^ ≈ [Cu(SF_5_phen)_2_]^2+^; this trend correlated well with that found for the half-wave potentials determined before.

Moreover, to better understand these nucleolytic activities, DNA affinity experiments were also carried out using EB displacement assays. The K_SV_ and K_app_ constants were determined, exhibiting the highest values for complexes [Cu(phen)_2_]^2+^ > [Cu(Fphen)_2_]^2+^, while the rest of the complexes exhibited lower binding affinities, most likely due to higher steric hindrances. On the other hand, evaluation of the nucleolytic activity in the absence of the reducing agent, complex [Cu(F_2_phen)_2_]^2^ was 2.5 times more active than [Cu(phen)_2_]^2+^, which had activity barely above that of the background (DNA reference). These results were plotted (% open circular DNA vs. half-wave potentials E_1/2_), producing a linear correlation. Thus, it was concluded that the more fluorine atoms incorporated in the ligand, the less negative the reduction potential observed for a given complex, hence enabling activation of the nuclease without an external reducing agent and leading to higher DNA cleavage rates.

In addition, cytotoxic experiments (using MTT assays) on MCF-7 breast cancer cells were performed. The cytotoxic activity of the complexes [Cu(CF_3_phen)_2_]^2+^, [Cu(SCF_3_phen)_2_]^2+^ and [Cu(SF_5_phen)_2_]^2+^ was almost the same compared to that of [Cu(phen)_2_]^2+^ in MCF-7 cells (IC_50_ 2.2–2.7 μm vs. 2.3 μm), while the complexes [Cu(Fphen)_2_]^2+^ and [Cu(F_2_phen)_2_]^2+^ exhibited lower cytotoxic activity. Similar experiments were also performed with fibroblasts (healthy cells), observing in this case that fluorinated complexes were less cytotoxic against fibroblasts than against MCF-7 breast cancer cells when compared to [Cu(phen)_2_]^2+^. Although there is no direct correlation between nucleolytic activity and DNA affinity, lipophilicity is thought to play an important role in the apoptosis process. That is, the complexes that contain the groups CF_3_, SCF_3_ and SF_5_, compared with those having F or F_2_, exhibited greater lipophilicity, directly influencing their cytotoxic activities. In fact, lipophilicity of the complexes was verified by water/n-octanol partition experiments, thus showing that incorporation of fluorinated groups can modulate the redox properties of the complexes, enhancing their biological properties.

**Scheme 2:**
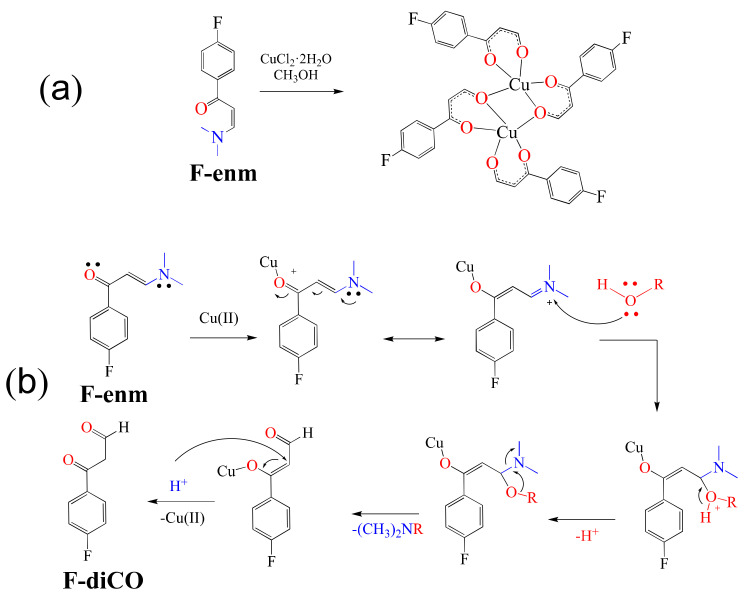
(**a**) Synthesis of complex [Cu_2_(F-diCO)_4_]. (**b**) Mechanism proposed for the transformation of the ligand F-enm into F-diCO.

Additionally, El-Ghamry et al. in 2021 reported the formation of fluorinated enaminone Cu(II) complexes [53]. In this regard, the authors mentioned that ligands play an important role in the cytotoxic abilities of the metal complexes. They chose to use enaminone ligands, a chelating type of ligand that in the presence of Cd(II) has previously shown interesting anticancer activities [54]. Thus, the fluorinated enaminone ligand F-enm was reacted with CuCl_2_·2H_2_O, forming the unexpected dinuclear complex [Cu_2_(F-diCO)_4_] (Scheme 2a), a product of the transformation of the enaminone into the 1,3-dicarbonyl ligand F-diCO (Scheme 2b).

With complex [Cu_2_(F-diCO)_4_] on hand, binding affinity experiments were carried out using salmon sperm DNA. Complex [Cu_2_(F-diCO)_4_] produced a decrease in the absorbance maximum (hypochromism), indicating that the affinity of the complex for DNA is the intercalation of the aromatic part of the F-diCO ligand through non-covalent interactions with the DNA base pairs. In addition, UV–Vis spectral titration served to determine the K_b_ value to be (3.62 ± 0.4) × 10^4^ M^−1^, revealing a moderate binding affinity when compared to that of the adduct EB-DNA (K_b_ = (1.23 ± 0.07) × 10^5^ M^−1^). Finally, the anticancer activity of the complex was evaluated against breast cancer cell line MCF-7 and the liver cancer cell line HepG-2. The IC_50_ values obtained were IC_50_ = 3.26 μg/mL for HepG-2 and 6.94 μg/mL for MCF-7, thus exhibiting higher activity compared with cisplatin (IC_50_ = 3.67 μg/mL for HepG-2 and 5.20 μg/mL for MCF-7).

## 5. Gold Metallodrugs

As mentioned above in Section 3, one of the advantages of the fluorinated compounds is their potential use in ^19^F magnetic resonance imaging (MRI) or ^18^F positron emission tomography [45]. However, a caveat is their low water solubility, a problem that can be sorted out by emulsification of these species into lipid micro/nanoparticle (NP) formulations.

**Figure 8:**
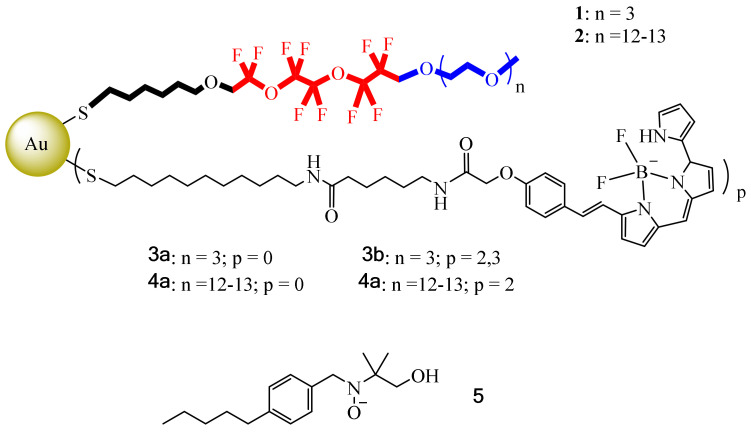
Thiolate ligands 1 and 2 without a dye. Thiolate ligands 3 and 4 bearing a dye (Bodipy). Small organic probe, molecule 5.

Nevertheless, such lipid formulations require long longitudinal (T_1_) relaxation times, a problem that can be solved by incorporating a Gd(III) ion to shorten T_1_. However, another difficulty with these lipid formulations is their large sizes (about 200–300 nm), which limit their imaging capabilities. So, in this context, Boccalon et al. sought to synthesize inorganic NPs using a metal center to serve as scaffold; in this case a gold surface was decorated with fluorinated ligands [55]. Here, the necessary qualities for ^19^F MRI or ^19^F NMR for therapeutic and diagnostic purposes were provided by the fluorinated ligands, ligands that must bear a dye to be detected by fluorescence. Another point to mention is the fact that these NPs must have the necessary physicochemical properties to avoid the use of surfactants. Furthermore, these molecular systems must have a size on an order of magnitude smaller than nanoemulsions, which may help to reach small vasculature vessels. In this case, Boccalon et al., inspired by the gold NPs reported by Niikura et al., designed similar gold NPs capped with fluorinated ligands (thiols 1 and 2) and coated them with a fluorescent dye, Bodipy 650/665 (ligands 3 and 4) (Figure 8). Noteworthy is the fact that in the fluorinated thiol used, the fragment near the surface of the gold was hydrophobic, and the central part of the ligand contained the fluorinated moiety, fundamental for the ^19^F signal for MRI, while the outermost part (triethylene oxide) was also relevant to impart water solubility. Due to the nature of these thiols, these NPs do not need additional amphiphiles for their solubilization/dispersion in water. However, for them to be detected by ^19^F MRI, they were also coated with thiols 3 and 4, which were tagged at the terminal part with a fluorescent dye, Bodipy 650/665. Once grouped in the gold center, these NPs could form diameter sizes from smaller than 2 nm to 10 nm in total.

In addition, MRI experiments were performed using complexes 3 and 4 (40 mg·mL^−1^) under a 7 T magnetic field, first tested against HeLa cells in uptake and cytotoxic activity. Then, it was determined by UV experiments that 3a and 4a had a very low number of fluorescent dyes per NP (2.3). Additionally, 3b tended to agglomerate in aqueous solution because the lipophilic part was too short compared to the hydrophobic Bodipy region. To overcome this problem, 4b was prepared including 2 Bodipy units per NP. With the compound on hand, cellular uptake and cytotoxic activity studies were performed with 4b using buffered solutions, adding salts with the aim of enhancing its aqueous solubility, while in the case of **5**, the ability of the monolayer to capture small organic probes was studied by electron spin resonance, with the results showing that **5** was inside the monolayer with the N-O group pointing to the fluorinated zone.

To further evaluate the cellular internalization of 4b in HeLa cells, confocal laser scanning microscopy (CLSM) analysis was used (2.5 mM of NPs for 4 h at 37 °C). The produced fluorescence images revealed the presence of 4b in the cell cytoplasm and its absence in the nucleus. Additionally, flow cytometry experiments were carried out, determining that 95% of the cells were viable after the uptake. This result indicates that 4b exhibited modest toxicity under the conditions tested. In this regard, the authors mentioned that despite the low cytotoxicity exhibited, these gold NPs were suitable imaging candidates with unprecedented versatility, highlighting the introduction of fluorine atoms as a very useful tool for MRI experiments.

## 6. Iridium Metallodrugs

**Figure 9:**
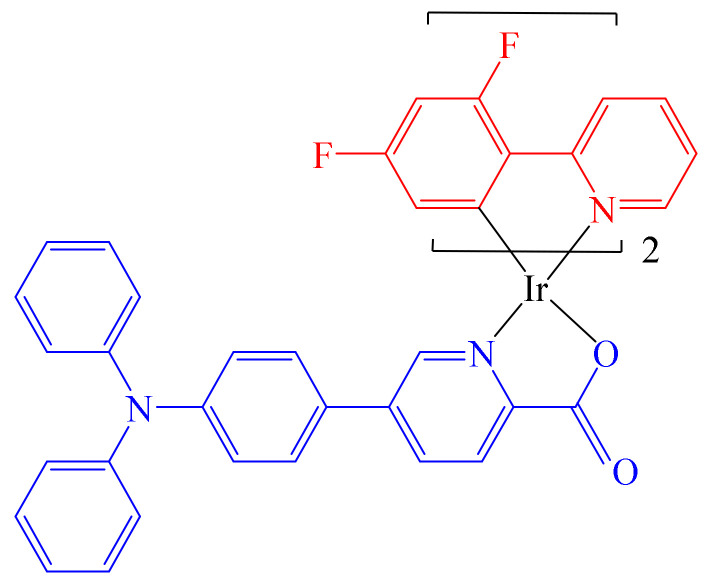
Ir (III) [(dfppy)_2_Ir(pic-TPA)] complex.

Few papers regarding the formation of iridium complexes with biomedical application have been reported. Most of the synthesis of these compounds has been focused on the preparation of dye-sensitized solar cells [56] and organic light-emitting devices (OLEDs) [57]. From structural-activity studies, it has been observed that during the chelate ligand design, the use of bulky heteroaromatic ligands is required, as this is a crucial point in the metal complex–DNA interaction [58]. In this regard, based on previously reported iridium complexes with efficient selectivity towards cancer cells, Bai et al. described the synthesis of compound [(dfppy)_2_Ir(pic-TPA)] with 2-(2,4-difluorophenyl)pyridine (dfppy) and triphenylamine tethered pyridine-2-carboxylate (pic-TPA), Figure 9 [58].

According to previous reports using analogous complexes, the use of bulky aromatic bonds with fluorine atoms decreases the possibility of ligand substitution reactions due to possible coordination with biomolecules before the complex–DNA interaction takes place.

Density functional theory (DFT) calculations showed that the incorporation of bulky substituents in the cyclometalated ligand (non-planar triphenylamine moiety) has a positive effect on the electroluminescent (EL) efficiency. Using spectroscopy studies, good quantum yield Φ_P_ = 0.8–1.0 and lifetime τ= 1.7–3.4 ns values were determined. Further, CLSM imaging results revealed complex [(dfppy)_2_Ir(pic-TPA)] to be found mainly in cell plasma and partially in the cell nucleus. In addition, antiproliferative experiments were carried out to compare the activity of [(dfppy)_2_Ir(pic-TPA)] with that of other compounds (Cisplatin, 5-Fluorouracil and Acyclovir) towards cancerous and normal cell lines. Here, Cisplatin and 5-Fluorouracil were used as positive control, and the Hek293 (human embryonic kidney) and Vero (African green monkey) cell lines were used as normal cell controls as well. The IC_50_ values determined for [(dfppy)_2_Ir(pic-TPA)] ranged between 4.38 ± 0.34 μM and 6.16 ± 0.14 μM, which were 4–18 times better than that of the reference Cisplatin (25.49 ± 1.2 μM to 108.18 ± 5.20 μM). In addition, the cytotoxic activity of complex [(dfppy)_2_Ir(pic-Br)] (19.49 ± 1.18 μM) with healthy cells compared with that of 5-Fluoroacil (10.33 ±1.05 μM) was reduced, with a selectivity ratio of cancer/normal cells of 1–2 orders of magnitude. Furthermore, radiosensitization activity of the compounds was evaluated using two cell lines, TE-1/TE-1R and Eca109/Eca109R, and the survival fraction was detected by colony formation assays. Determination of the sensitization enhancement ratio (SER) showed compound [(dfppy)_2_Ir(pic-Br)] to have a more pronounced decrease than complex [(dfppy)_2_Ir(pic-Br)]. Thus, cell cytometry experiments showed that complex [(dfppy)_2_Ir(pic-TPA)] arrested cells in G2 phase (27.82% vs. 11.75% in blank control and 13.03% with Cisplatin), whereas treatment with complex [(dfppy)_2_Ir(pic-Br)] produced a G2 arrest of only 22.68%, thus explaining the lower SER value found in the radiosensitization effect experiments. Molecular docking calculations allowed the determination of the estimated binding energies towards DNA of −8.9 kcal × mol^−1^ for [(dfppy)_2_Ir(pic-Br)] and of −9.6 kcal × mol^−1^ for [(dfppy)_2_Ir(pic-TPA)], clearly indicating the better affinity of [(dfppy)_2_Ir(pic-TPA)], thus impacting directly the interference with the cancer cell nucleic acid replication phase. According to the authors, complex [(dfppy)_2_Ir(pic-TPA)] represents an ideal candidate when drug resistance occurs, with applicability in radio chemotherapy and combination therapy.

**Figure 10:**
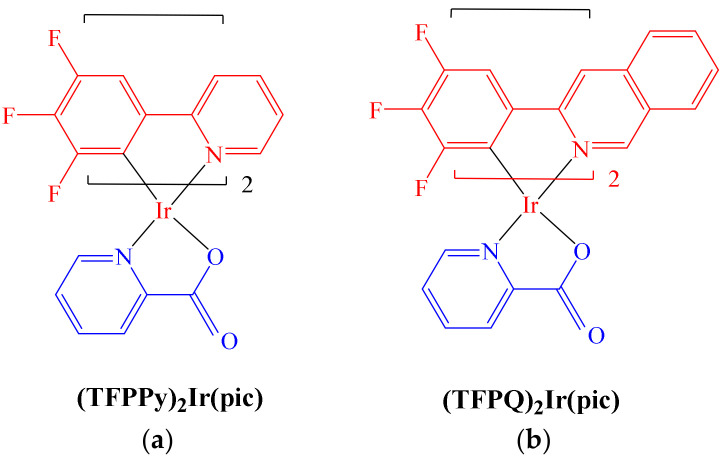
Ir (III) [(TFPPy)_2_Ir(pic)] (**a**) and [(TFPQ)_2_Ir(pic)] complexes (**b**).

Following their previous studies, in 2020 Bai et al. reported the formation of a series of novel Ir(III) complexes including fluorinated ligands inspired by the auxiliary ligand 2- (2,4-difluorophenyl) pyridine (dfppy); hence, some modifications were made to produce the ligands 2- (3,4,5-trifluorophenyl) pyridine (TFPPy) and 2-(3,4,5-trifluorophenyl) quinoline (TFPQ). With these fluorinated ligands on hand and in the presence of pyridine-2-carboxylate, the Ir(III) complexes [(TFPPy)_2_Ir(pic)] and [(TFPQ)_2_Ir(pic)] were synthetized (Figure 10) [59]. The idea behind these new ligands was to provide resistance to ligand substitution reactions, thus avoiding the binding of some biomolecules before complex–DNA interaction occurred.

Images obtained by CLSM experiments proved that both complexes resided mainly in cell plasma due to hydrophobic interactions between the aromatic moieties with the phospholipidic bilayer and cytoplasmic proteins. In addition, molecular docking calculations revealed the binding energies for [(TFPQ)_2_Ir(Pic)] and for [(TFPPy)_2_Ir(Pic)] to be −8.9 kcal·mol^−1^ and −8.4 kcal·mol^−1^, respectively. According to the authors, the complexes adopt a conformation to bind to the main groove of the nucleic acid helix, such that the pyridine and quinoline fragments do not greatly affect their binding activity against the viral nucleic acid strands. Additionally, IC_50_ assays with both complexes were performed using diverse cancer and healthy cell lines, with Cisplatin and Fluorouracil as positive controls. Results obtained showed that in general, the complexes exhibited selectivity against various cancer cell lines (1–2 orders of magnitude). In this paper, the radiosensitization activity of both complexes in the radiation-resistance sublines Eca109 and Eca109R was also assessed. SER values were observed in the range of 1.74–1.94 for both complexes, values that, when compared with other reported radiotherapy sensitizers such as Resveratrol (1.28), Paclitaxel (1.32), gold nanoparticles (1.19), α-cyanostilbene (1.62) and trifluorinated Iridium complexes, [59] revealed both complexes [(TFPQ)_2_Ir(Pic)] and [(TFPPy)_2_Ir(Pic)] to have enhanced SER values. For radio-resistant cell lines after treatment, the suggested mechanism proceeds through the typical arrest of the G2 phase. By means of flow cytometry experiments, it was determined that apoptosis was dose-dependent. Furthermore, both complexes exhibited antiviral effects towards HSV-1 and HSV-2 virus strands. Overall, the authors concluded that both complexes could be considered as suitable substitutes when conventional [Pt(NH_3_)_2_X_2_] drug resistance occur.

## 7. Platinum Metallodrugs

**Figure 11:**
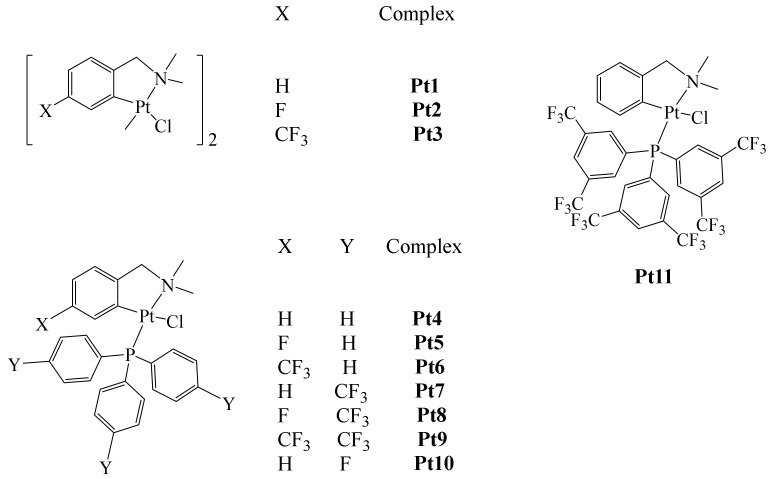
Pt(II) Pt1–Pt11 fluorinated complexes.

As mentioned above, despite their observed benefits as antitumor agents, Pt-based metallodrugs also exhibit several undesired side effects, which is the reason similar complexes have been synthesized. In this line, nonconventional C,N-cycloplatinated(II) phosphine antitumor complexes were prepared by Cutillas et al. (Figure 11) [60]. According to the authors, only complexes Pt4–Pt11 were evaluated in biological studies. Cytotoxic experiments were carried out towards T47D human breast cancer cell line and epithelial ovarian carcinoma cells A2780 and A2780cisR. Nonfluorinated complex Pt4 was used for comparison purposes. The results obtained showed complexes Pt7–Pt9 to exhibit the best cytotoxic effect in all tested cell lines. Complex Pt7 was particularly active against T47D, with an IC_50_ value 16 times smaller than that of cisplatin and 11 times smaller than that of the non-fluorinated analog [Pt(dmba)(PPh_3_)Cl] (dmba = dimethylaminomethyl-phenyl). Further, the Cisplatin resistance factor (RF) against A2780cisR (cisplatin-resistant ovarian carcinoma) was also determined, finding values of RF for complexes Pt4, Pt6, Pt8–Pt11 in the range of 0.5–1.0; for Pt7 only, a value above 2 was determined (an RF < 2 implies no cross resistance, indicating circumvention of cisplatin resistance).

Following this work, cell cycle arrest studies were performed only with compounds Pt7, Pt8, and Pt9 (at previously determined IC_50_ concentrations) using the DNA dyed with propidium iodide (PI) protocol. The three complexes showed a slight enhancement in G0/G1 phase with respect to control cells (1.8%, 4.2%, and 6.4%). Cell cycle arrest in the G0/G1 phase contrasted with the previously published effect of Cisplatin [61]. In addition, analysis by flow cytometry experiments showed that treatment of A2780 cell lines with complex Pt9 strongly induced apoptosis in these cancer cells. Moreover, using these same cells, platinum accumulation was determined to be approximately 50 ng/10^6^ cells after 240 min of incubation; comparison with that of Cisplatin (0.71 ± 0.13 ng/10^6^ cells in the same conditions) showed that complex Pt9 had a stronger ability to penetrate into A2780 cells. Thus, the authors demonstrated that complexes Pt7−Pt9 containing P(C_6_H_4_CF_3_-p)_3_ exhibited superior cytotoxic activity against the tested cancer cell lines compared with Cisplatin.

**Scheme 3:**
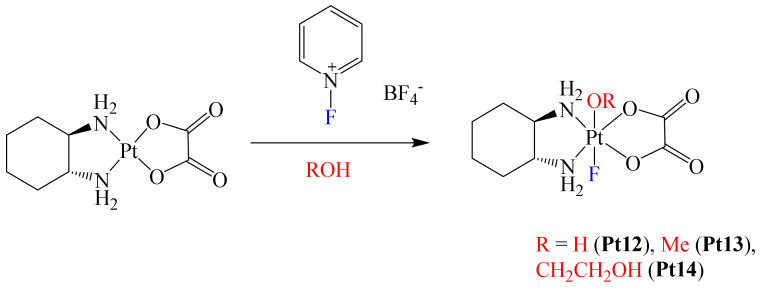
Pt(IV) Pt12–Pt14 fluorinated complexes.

After this work was published, Xu et al. reported the structural modification of the metallodrug oxaliplatin. In this case, they prepared a series of novel Pt(IV) complexes bearing an axial fluoride ligand (Scheme 3) [62]. This was based on the growing interest in the synthesis of Pt(IV) complexes, due to their promising clinical properties [4].

In this paper, the authors mentioned that the physicochemical and biological properties of some Pt(IV) metallodrugs may be affected depending on the nature of the axial ligand. Authors foresaw this with the axial incorporation of a fluorine atom. Electrochemical experiments showed that fluorination at the axial position of the complexes induces more negative reduction potentials, decreasing their reduction reaction rate by ascorbate. However, the presence of the fluorine atom did not enhance the lipophilicity of the complexes; in fact, all complexes were hydrophilic. Apparently, the lack of hydrophobic carbon–fluorine bonds in the complex limited the increase in lipophilicity [63]. Additionally, cytotoxic essays using complexes Pt12–Pt14 towards A2780 and A2780cisR cell lines afforded the following trend: Pt12 > Pt14 > Pt13 for both cell lines. To have a better understanding of the permeation effect due to complex fluorination, platinum accumulation in A2780 and A2780cisR cells was determined. These results were compared with those of oxaliplatin, with observation of the following trend of degree of accumulation in A2780 cells: oxaliplatin > Pt12 > Pt14 > Pt13. This trend was consistent with the cytotoxic results obtained, with oxaliplatin exhibiting the lowest IC_50_ value because of a higher permeation effect (highest cellular accumulation level). Thus, cellular accumulation was considered as a direct factor for cytotoxicity. In the case of the A2780cisR cells, only complex Pt12 exhibited a decreased level of cellular accumulation compared with that observed in A2780 cells. Based on the above, it was concluded that one of the benefits on introducing fluorine in these complexes was their good stability under physiological conditions, although their cytotoxicity was lower compared to that of oxaliplatin, thus leaving further opportunities for a more adequate optimization in the design of the ligands to further improve their delivery capacity.

**Figure 12:**
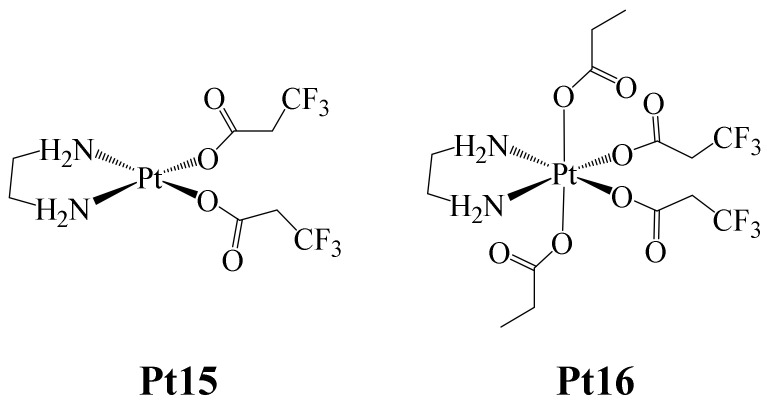
Pt (IV) Pt15–Pt16 fluorinated complexes.

On the other hand, Höfer et al. reported in 2019 the formation of the Pt(II) and Pt(IV) complexes shown in Figure 12 (Pt(IV) complexes can be designed as prodrugs, which have to be reduced to have a proper anticancer activity) [64]. The interest in the design of Pt(IV) complexes as prodrugs is due to the fact that they are kinetically inert and are not very prone to ligand substitution. Their activation as cytotoxic agents (reduction to Pt(II)) takes place inside the cancer cell). In this way, if the Pt(IV) prodrug is resistant to reduction, its anticancer activation can be controlled until it is reduced to Pt(II), which prevents unwanted side effects from occurring.

Here, complex Pt15 was synthesized starting from the complex dichlorido(ethane-1,2-diamine) platinum (II), to which two 3,3,3-trifluoropropanoate (tfpa) ligands were coordinated. Additionally, complex Pt16 was prepared by the oxidation of complex Pt15 with H_2_O_2_ and further treated with acetic anhydride.

Both complexes were tested against the human cancer cell lines CH1/PA-1 (ovarian teratocarcinoma), SW480 (colon carcinoma) and A549 (non-small cell lung carcinoma). The trend found in the IC_50_ values for both complexes was: CH1/PA-1 < SW480 < A549. The IC_50_ values determined for Pt15 (CH1/PA-1 (0.67 ± 19 μM), SW480 (11 ± 2 μM) and A549 (26 ± 6 μM)), ranged in concentration between those of Cisplatin and Carboplatin, which were used as positive controls. In the case of Pt16, the IC_50_ values were lower (CH1/PA-1 (1.1 ± 0.2 μM), SW480 (14 ± 3 μM) and A549 (18 ± 4 μM)), this being attributed to the presence of monodentate carboxylate ligands, which facilitate rapid ligand exchange reactions such as those occurring with chlorides. The opposite was observed with Carboplatin, which is bound to a bidentate dicarboxylate ligand (Figure 2), and showed higher IC_50_ values due to its greater inertness (CH1/PA-1 (1.4 ± 0.4 μM), SW480 (85 ± 28 μM) and A549 (91 ± 10 μM)). The IC_50_ values found for Pt15 and Pt16 were similar in all cancer cell lines, probably due to the fact that the Pt(IV) complex can be reduced, giving rise to the Pt(II) species, ultimately producing a higher cytotoxic effect because in the mechanism of action of Cisplatin, Pt(II) must undergo aquation, and this species reacts directly with DNA (Pt-DNA adduct), inducing apoptosis. Therefore, once inside the tumor, the Pt(IV) complex must be reduced, and the aqua complex immediately formed. Based on this, the intracellular reduction behavior of the Pt16 complex was estimated. Thus, Pt16 complex reduction experiments were carried out in lysates of SW480 and CH1/PA-1 cancer cells and were monitored by ^19^F NMR. The results showed that the 50% reduction in Pt16 occurred after 27 h for the chemosensitive cell line CH1/PA-1, taking about one third of that time (~10 h) for the resistant cell line SW480. These findings, compared with those previously observed with the carboplatin tetracarboxylate analogue [65], showed that complex Pt16 reduces faster, since the other species required about 100 h for a 50% reduction. In this way, the authors demonstrated that the nature of the ligand is important in Pt(IV) complexes in making the reduction process faster, facilitating further aquation, and finally leading to apoptotic cell death.

**Figure 13:**
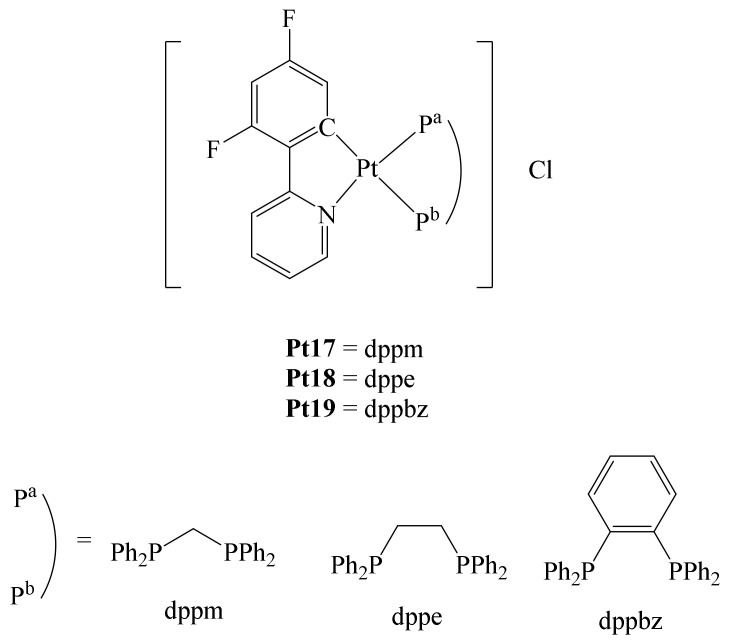
Pt (IV) Pt17–Pt19 fluorinated complexes.

Similarly, Shahsavari et al. reported the preparation of a series of cycloplatinated (II) complexes bearing different diphosphines and the ligand 2-(2,4-difluorophenyl)pyridine (dfppy) (Figure 13) [66]. They were interested in this ligand due to its chelating nature and the strong Pt-C bond formed, which can provide stability to the complex under physiological conditions to reach the desired target.

Furthermore, the incorporation of the diphosphines ((diphenylphosphino)methane (dppm), (diphenylphosphino)ethane (dppe) and 1,2-bis(diphenylphosphino)benzene (dppbz)) in the complexes conferred higher lipophilicity, enhancing the cytotoxic ability of these compounds. In addition, it has been described that cationic cyclometalated Pt(II) complexes may have a higher cytotoxicity than Cisplatin in HCT-116 colon cancer cells (148-fold increase) [67]. The authors anticipated that the hydrophobicity of these complexes would be modified with the fluorination of the ligand dfppy. With these ideas in mind, complexes Pt17–Pt19 were synthesized (Figure 13). By different techniques, it was determined that complexes Pt17–Pt19 have good stability in physiological media. The cytotoxicity of complexes Pt17–Pt19 was evaluated in vitro towards different human cancer cell lines, e.g., cervical cancer (Hela), carcinoma of the ovary (SKOV3) and breast (MCF-7), as well as a non-tumor cell line (MCF-10A; normal human epithelium mammary cell line). In comparison to Cisplatin, the three complexes Pt17–Pt19 exhibited higher antiproliferative activity on the cell lines studied, with complex Pt19 exhibiting the best antiproliferative abilities. In addition, the selectivity of these complexes towards cancer and normal cell lines was evaluated. Again, all complexes showed better selectivity towards all cell lines tested compared with Cisplatin. Notably, the three complexes were also tested by the National Cancer Institute against 60 different human tumor cell lines such as leukemia, melanoma, lung, colon, brain, ovary, breast, prostate, and kidney. Thus, against cell lines such as K-562 (leukemia), HOP-92 (lung), HCT-116 (colon), OVCAR-8 (ovarian), PC-3 (prostate), MDA-MB-468 (breast), and melanoma, complexes Pt17–Pt19 exhibited higher cytotoxicity than Cisplatin. Additionally, docking calculations revealed that complex–DNA interactions occur through hydrophobic interactions with the base pairs in the minor and major grooves of DNA. Of the three complexes, compound Pt19 showed the highest antiproliferative activity towards any of the cell lines tested, thus positioning this series of complexes as potentially promising metallodrugs in the treatment of cancer.

**Figure 14:**
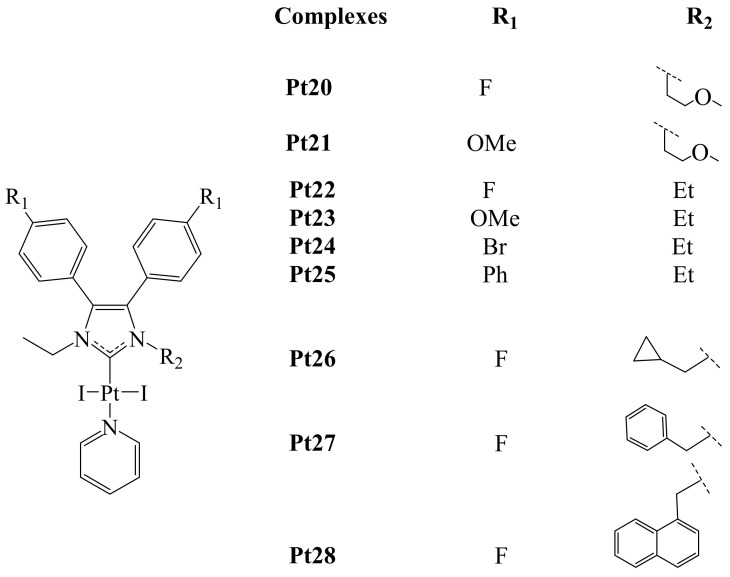
Pt(II)-NHC Pt20–Pt28 complexes.

**Figure 15:**
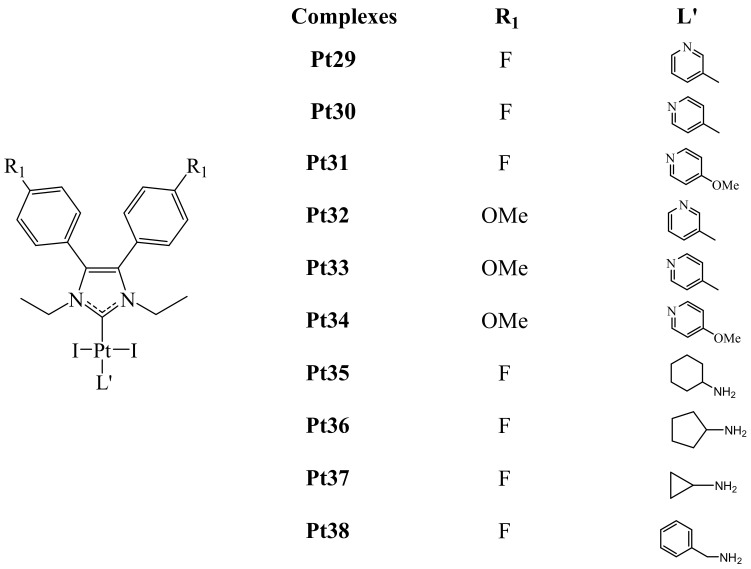
Pt(II)-NHC Pt29–Pt38 complexes.

Recently, Bian et al. reported the synthesis of a series of Pt(II)-NHC complexes. With the goal of prepare metal complexes able to act simultaneously as chemo- and immunotherapeutic agents [68]. In this context, platinum-derived complexes have these characteristics. Including reports of Pt(IV) prodrugs, which can stimulate the proliferation of T cells and simultaneously induce programmed apoptosis of cancer cells. Besides, the authors mentioned at least three other different types of Pt(II) and Pt(IV) complexes that meet these two characteristics. However, they highlighted that complexes of the type Pt(II)-NHC have shown interesting in vitro activities, mainly in the treatment of hepatocellular carcinoma (HCC). HCC represents a very lethal malignancy, with a high incidence of cases and poor prognosis. In addition, current therapies for its treatment are not effective. Interestingly, these authors and other groups had previously published the preparation of different M-NHC complexes derived from 4,5-diarylimidazole (M = Ag(I), Au(I/III) and Rh(I)), exhibiting excellent anti-HCC activities in vitro [69,70,71,72,73]. Hence, Bian et al. reported the synthesis of a series of Pt(II)-NHC compounds derived from 4,5-diarylimidazole (Figure 14 and Figure 15) [68].

The antiproliferative activity of these complexes was evaluated in vitro using the MTT assay. Among all the complexes, fluorinated complex Pt22 exhibited the best anticancer activity towards HT-29, MFC-7, A2780, A549, HepG2 and Hepa-6 cancer cells. Being better than that of Cisplatin. Besides, based on structure-activity relationship studies, it was concluded that the 4-OMe or 4-F substituted complexes presented better activity than the rest of the series. Thus, complexes Pt26 and Pt28 showed modest antitumor activity even with the introduction of more lipophilic groups (cyclopropyl and naphthyl) at the N-substituent. Additionally, in the case of complexes Pt29–Pt34, the presence of electron-donating groups at the pyridine did not have a great effec on their anticancer activity.

The authors emphasized that many Pt-based metallodrugs can promote immune system activation that favors treatment against certain types of cancer. In this case, immunogenic cell death (ICD) is associated with the release of damage-associated molecular pattern signals (DAMPs). During ICD, the release of large amounts of HMGB1 and ATP into the extracellular space was detected. Thus, the presence of HMGB1 is considered a late apoptotic marker indicating the release of tumor antigens to dendritic cells.

Besides, studies in Hepa1-6 cells did not show that Cisplatin induced the production of the calcium storage protein calreticulin (CRT), however, a high expression of CRT was observed when Hepa1-6 cells were treated with complex Pt22 and Oxaliplatin. These results were simultaneously corroborated by cytometry analysis and the use of confocal microscopy. The liberation of CRT drives the activation of antigenic cells, which intracellularly represents the intensity of DAMPs release. In this sense, complex Pt22 induced CRT translocation more efficiently than Oxaliplatin. Additionally, determination of ATP levels in the culture medium allowed to see that complex Pt22 (33.5 nM) induced higher quantities of ATP than Oxaliplatin (26.0 nM). Translocation of HMGB1 from the nucleus to the cytoplasm was also evaluated. The assays with DMF as control and the cells treated with Cisplatin showed that HMGB1 remained in the nucleus, the opposite in the case with complex Pt22 and Oxaliplatin, where this marker moved from the nucleus to the cytoplasm. Thus, allowing to conclude that both complex Pt22 and Oxaliplatin are ICD inducers since they stimulate the activation of HMGB1, CRT and ATP biomarkers. While Cisplatin does not, at least in HCC cancer cells.

Additionally, using inductively coupled plasma-mass spectrometry, the authors demonstrated that complex Pt22 accumulates in mitochondria, by observing, an altered mitochondrial morphology, an increased glutathione/glutathione disulfide ratio, and increased superoxide dismutase levels all indicative of ROS accumulation and mitochondrial dysfunction in HepG2 treated cells.

Further, DCFH-DA staining experiments demonstrated the induction of ROS in complex Pt22 treated cells and endoplasmic reticulum tinction colocalization suggested the involvement of this organelle in cell apoptosis. In the same line, Hepa1-6 cell death induction was repressed when specific inhibitors of the ERS signaling or the antioxidant NAC were added to the media, suggesting that DC activation probably requires the cytotoxic effect of ROS through ERS signaling pathways. Accordingly, complex Pt22 treated Hepa1-6 cells exhibited increased markers of apoptosis derived of ERS (CHOP and calnexin), and co-cultivation with DC revealed increased markers of maturation of these immune cells.

On the other hand, UV/vis shift spectra and circular dichroism assays suggested that compound Pt22 binds to the minor groove of DNA. And produces double-strand breaks probably triggering apoptosis through the expression of p53. Besides, propidium diiodide tinction and D1 and CDK4 downregulated levels suggested that complex Pt22 arrests the cells in the S phase. Promoting apoptosis through the intrinsic pathway, as reflected by cytochrome c, Bak, Bax and cleaved caspase 3/9 upregulation in HepG2 Pt22 treated cells.

To further validate that complex Pt22 promotes ICD in vivo, a tumor vaccination murid model was used, and the results indicated that the CRT levels (the main characteristic of ICD) were upregulated in tumor tissues when compared to control. The tumor growth was repressed in the complex Pt22 treated group and did not show significant alterations in other tissues (heart, liver, lung, kidney), an effect that highlights the specificity of this compound for tumor cells.

On the other hand, a CCl_4_-induced liver injury model showed that complex Pt22-treated mice bear lower levels of ALT and AST and reduced fibrogenesis when compared with control. Furthermore, analysis of DC and T cells in the spleen showed increased numbers of both cells in complex Pt22-treated groups, indicating that this compound increases the proliferation rate of immune cells when compared to control.

In conclusion, complex Pt22 triggers immune function through the activation and proliferation of DC, and this effect is derived from DNA damage and RES in tumoral cells.

**Figure 16:**
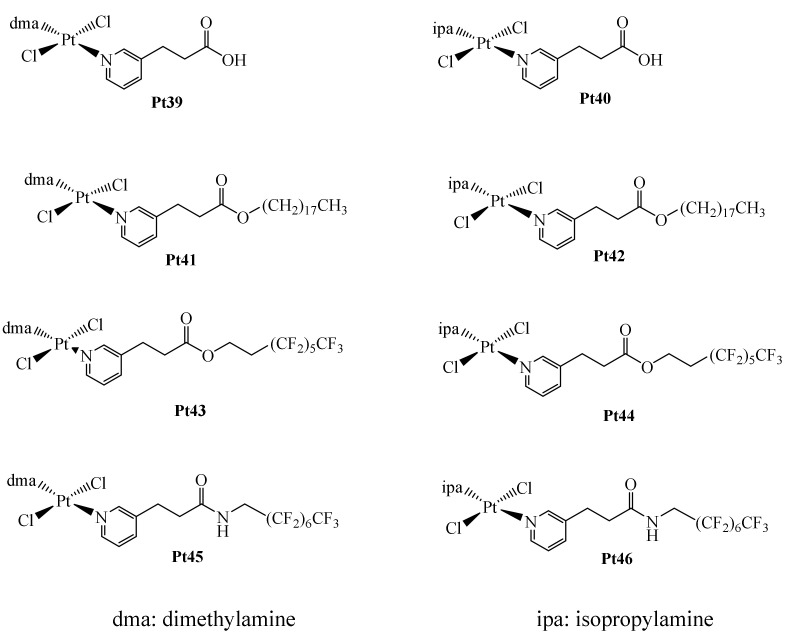
Thermoresponsive Pt(II)-complexes containing perfluorinated chains Pt39–Pt46.

Recently, in the use of Pt-based metallodrugs, there has been an interest on their use, simultaneously with another therapeutic method since they are usually more effective in this manner, than when used as monotherapies. For example, in the treatment of retinoblastoma, the simultaneous combination of Carboplatin and selective heating of the tumor in question (thermotherapy) is substantially more effective than the two monotherapies alone. Because of this, there has been an enormous interest in the design of Pt metallodrugs with thermoresponsive properties. In this case, there is a thermoactivation of Carboplatin after selective heating of the tumor. In this context, Cabrera et al. designed a series of Pt(II) complexes including perfluorinated chains since they can confer thermoresponsive capabilities to the complexes (Figure 16) [74]. In addition, they synthesized another series with long non-fluorinated alkyl chains for comparison. Being complexes Pt39–Pt40 the precursors to produce the series of complexes Pt41–Pt46.

The complexes shown in Figure 16 were evaluated for their interaction with supercoiled pBR322 plasmid DNA. Initially, complexes Pt39–Pt40 were tested with DNA. Complex Pt39 interacts with the ccc form of the supercoiled DNA, presenting a r_i_ value of 0.1, while complex Pt40 of 0.2. However, raising the temperature of the experiment did not cause an increase in the interaction of these complexes with DNA. Similarly, no interaction of complex Pt41 with DNA was observed, even by raising the temperature to 42 °C. This lack of interaction is attributed to the low solubility of complex Pt41. On the other hand, complex Pt42 showed interaction ability like that of its precursor, Pt40. Being the poor interaction of complexes Pt39–Pt42 with DNA due to the absence of the perfluorinated chains, since the series of complexes Pt41–Pt46 exhibited more interesting results. In addition, the results of the complex–DNA interaction with complexes Pt43–Pt44 showed that complex Pt44 had a stronger interaction. This result being consistent with what was observed in the determination of the stability constants in DMSO, where complex Pt43 is less stable in solution, indicating that complex Pt44 is more reactive towards DNA. And complexes Pt45–Pt46 exhibited greater interactions with DNA. This being attributed to the fact that they contain the longest perfluorinated chains, which increases their reactivity towards DNA and thus, greater cytotoxicity. Further, cytotoxicity studies of the series of complexes Pt39–Pt46 were carried out on the cancer cell lines A2780 (human ovarian carcinoma) and A2780cisR (human ovarian carcinoma with acquired resistance to Cisplatin). And the HEK293 non-tumorigenic (human embryonic kidney) cell line. Using concentration of the complexes at 100 μM, the series of complexes Pt39–Pt41 did not show relevant cytotoxic effects; however, complex Pt42 exhibited the better activity but not above that of Cisplatin. In the case of the complexes with perfluorinated chains Pt43 and Pt45–Pt46 they showed the best cytotoxic effects. Albeit with no cell selectivity. Additionally, thermoresponsive experiments with all the complexes were carried out, however, no synergistic effect was observed by the combination of therapies, although at higher temperatures the perfluorinated compounds exhibited the best results.

In addition, protein-binding metallodrug interactions with bovine pancreatic ribonuclease A were determined. These experiments were carried out at 37 and 42 °C, and all determinations were assumed to be pseudo first-order reactions. However, the experiments at 42 °C did not show a clear trend in the results, likely due to protein denaturation at these temperatures. And, in the experiments at 37 °C, the non-perfluorinated complexes showed certain affinity towards the protein. The complex precursors Pt39–Pt40 exhibited the lowest values, and the complexes Pt41–Pt42 showed the best affinity presumably due to interactions of the aliphatic chains with the lipophilic part of the protein. Finally, the perfluorinated complexes presented the highest affinity values, indicating that there are many interactions due to the presence of the fluorine atoms. Thus, in summary, the perfluorinated complexes presented the best cytotoxicity and affinity capabilities; however, they did not exhibit relevant thermoresponsive properties nor cancer cell selectivity.

## 8. Ruthenium Metallodrugs

**Figure 17:**
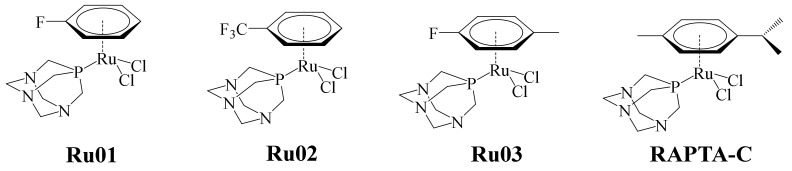
Ru(II) Ru01–Ru03 fluorinated complexes.

In recent years, ruthenium complexes have been considered as a potential alternative to platinum-based chemotherapeutic agents. In particular, the ruthenium (II)-arene (RAPTA) complexes (pta = 1,3,5-triaza-7-phosphaadamantane) have exhibited relevant toxicity towards metastatic tumors in in vitro and in vivo studies. The selectivity of these complexes apparently is pH-dependent. Typically, the pH of tumor cell medium is lowered to 5.5, and the healthy cell environment is about 7.2. In this sense, it has been said that this type of complex exerts significant unwinding to supercoiled DNA at pH values below 7.0, without affecting the DNA. Taking into consideration the pH difference scenario between tumor cells and healthy ones, a series of complexes were designed by means of DFT calculations. In the pH conditions of tumor cells, the aqua complexes must prevail, contrary to the pH in healthy cells, where the less reactive hydroxo compounds must dominate. Thus, according to the calculations, the incorporation of electron-withdrawing substituents on the arene in RAPTA complexes can modulate the equilibrium between the hydroxo- and aqua-complexes. RAPTA-C remains the best anticancer metallodrug of this series (Figure 17) [75]. The new, modified RAPTA complexes designed by Renfrew et al. are shown in Figure 17 [76].

The cytotoxic activity of complexes Ru01–Ru03 was evaluated against the A2780 human ovarian cancer cell line, using RAPTA-C as positive control (Figure 17). In general, Ru02 and Ru03 showed higher antiproliferative effects on the A2780 cell line compared with RAPTA-C. The less cytotoxic complex of the series was Ru01, this being because the arene ligand is more labile as a consequence of its substituents. Previous studies of the binding of RAPTA-C to an oligonucleotide showed that a key step in this process was the loss of the ligand arene. This step causes distortions in the oligonucleotide due to increased coordination demands of the complex. Although Ru02 and Ru03 had better cytotoxic activity than RAPTA-C, the latter was much more effective in in vivo tests against metastatic and primary tumors (at high doses). Meanwhile, due to the higher cytotoxicity shown by Ru02 and Ru03, they are clinically applicable at lower doses. This is of great interest from a pharmacological point of view.

**Figure 18:**
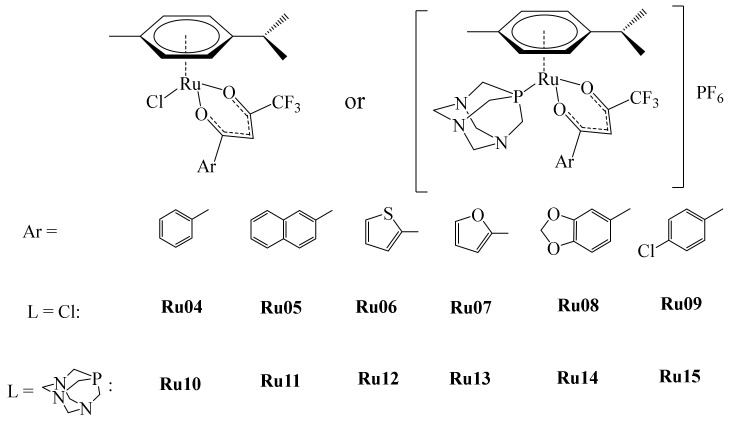
Ru(II) Ru04–Ru15 fluorinated complexes.

Subsequently, in 2015, Seršen et al. reported the preparation of some novel ruthenium-based metallodrugs. Again, based on the RAPTA series, this time the authors proposed to modify the main scaffold, incorporating fluorinated diketonate ligands (Figure 18) [77].

Notably, complexes Ru04–Ru09 contain a Ru(II)-Cl bond that is known to be labile, permitting the formation of the Ru(II)-OH_2_ species in aqueous solutions, necessary for the binding towards biomolecules such as proteins and/or DNA [78]. Moreover, Ru(II)-Cl complexes under high Cl^−^ concentrations in culture cell media (>100 mM) or the bloodstream (~100 mM) are relatively stable. Activation of these species in intracellular space is initiated at low Cl^−^ concentrations (~4 mM). For this reason, the authors prepared complexes Ru10–Ru15 by replacing the Cl ligand with pta to prevent hydrolysis. It has been reported that the pta ligand in RAPTA-C is involved with the c-jun N-terminal kinase (JNK) mediating G2/M phase cell cycle arrest and apoptosis via activation of the tumor suppressor p53. Additionally, the cationic nature of these complexes enhances their water solubility. The electron-withdrawing -CF_3_ group appended to the diketonate ligand enhances lipophilicity, improving cellular uptake. The aquation process in the Ru(II)-Cl complexes was evaluated by ^1^H NMR in order to determine their stability, finding that the hydrolysis of complexes Ru04–Ru09 forming the ionic key step species [(η^6^-p-cymene)Ru(κ^2^-O,O-β-diketonate)(OH_2_)]^+^ occurs rapidly. After 24 h in aqueous solution, the formation of the diketonate-free species [(η^6^-p-cymene)Ru_2_(OH_2_)_3_]^+^ was observed. These dinuclear hydrolyzed ruthenium species are known to be thermodynamically stable and unreactive. The opposite happened with the series of complexes Ru10–Ru15, which remained stable after being in aqueous solutions for more than 48 h, only showing minor structural changes (δ 7.21−7.27 ppm). All of these metallodrugs were tested in vitro towards ovarian cancer (CH1) and osteosarcoma (MG63) cell lines. Further, to estimate their selectivity, the complexes were evaluated against a nonmalignant keratinocytes-derived cell line (HaCaT) under similar conditions. In general, the cell HaCaT was resistant to all of the complexes except Ru15, which exhibited an IC_50_ value of 72 μM. The CH1 cell line in most of the cases was more sensitive compared with the results with MG63 cells. From the results, the authors were able to establish a structure-activity relationship. CH1 cells were more sensitive towards the Ru(II)-Cl complexes than to the Ru(II)-pta derivatives. However, osteosarcoma MG63 cells were more sensitive to Ru(II)-pta derivatives. In the cellular accumulation experiments of these complexes, it was observed that the Ru(II)-Cl derivatives (Ru08 and Ru09) entered the cancer cells more efficiently compared to their pta counterparts (Ru14 and Ru15), where this effect was much lower. The authors hypothesized that this lower effect of cell accumulation is due to steric effects, in contrast to the case with the Ru(II)-Cl derivatives, where the chlorido ligand is released, facilitating the interaction and cell uptake. In the cell viability studies, the Ru(II)-pta derivatives, despite having a greater impact on this effect, were outperformed by the chlorido analogues, which showed better results in cell accumulation in adherent and nonadherent malignant cells. In general, chlorido compounds exerted oxidative stress, contrary to the Ru(II)-pta counterparts in which ROS induction was less prominent, with the exception of complex Ru15. In the evaluation of the phases of the cell cycle, the Ru(II)-pta compounds induced accumulation in the G0/G1 phase, contrary to the chlorido analogues, which acted in the G2/M phase. It should be noted that this G2/M phase is also characteristic for the DNA-damaging compound Cisplatin. Based on these results, the authors noted that research should continue to seek a fine tuning of the chemical structure that allows modulation of the anticancer properties of these species.

**Figure 19:**
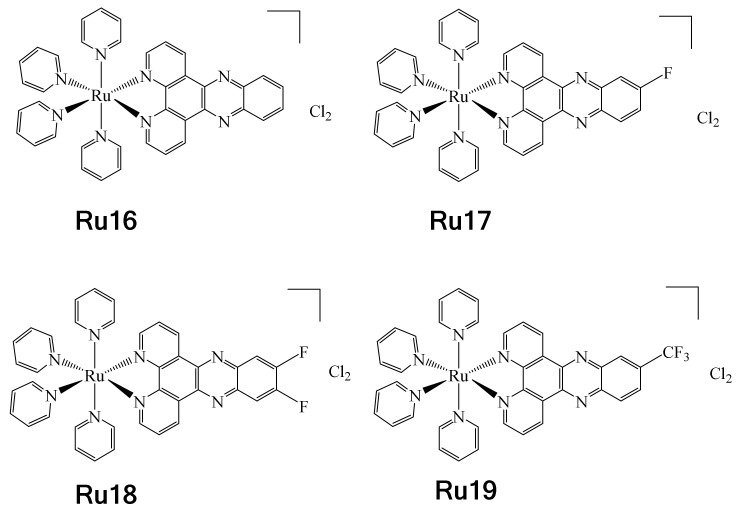
Ru(II) Ru04–Ru15 fluorinated complexes including fluorinated dppz ligands.

On the other hand, in 2017, Boerhan et al. reported the synthesis of a series of Ru complexes containing three different types of fluorinated ligands, i.e., **F**-dppz = 11-fluoro-dipyrido [3,2-a:2′,3′-c] phenazine, F2-dppz = 11,12-difluorodipyrido [3,2-a:2′,3′-c] phenazine and CF_3_-dppz = 11- (trifluoromethyl)-dipyrido [3,2-a:2′,3′-c] phenazine [79]. These complexes were synthesized with the aim to be used in photodynamic therapy (PDT) and photoactivated chemotherapy (PACT) [80,81]. These types of therapies are usually less invasive and may have fewer side effects. It should be noted that dppz = (dipyrido[3,2-a:2′,3′-c]phenazine) is a well-known DNA intercalating ligand (Ru16), and precisely for this reason the authors used it as a model and synthesized fluorinated analogues to evaluate their effect on PACT (Ru17–Ru19) (Figure 19). The UV-vis properties of complexes Ru16–Ru19 were determined, showing luminesce at 77 K. It was observed that as the number of fluorine atoms in the complexes increased, there was a redshift from 605 nm for Ru16 to 616 nm for Ru19. This was attributed to the greater electronegative effect conferred by the fluorine atoms, leading to red-shifted MLCT emission. These complexes exhibited rapid changes in absorption when LED-irradiated at 470 nm and were stable in the dark. It was observed that during irradiation, complex Ru18 underwent a transformation to a new product (exchange of pyridine for a Cl^−^), which was corroborated by HR ESI-MS. Additionally, titration experiments allowed the determination of the complex–DNA affinity constants. From these results, it was determined that complexes Ru16 and Ru17 presented similar affinity, while complex Ru18 showed a considerable increase and Ru19 experienced a noticeable decrease. The disparities found in the affinity constants were attributed to the effects of hydrophobicity. However, this hypothesis was discarded when the values of log P_o/w_ were determined. So, the authors believe that in the specific case of the difference in affinity constants between complexes Ru17 and Ru18, this is due to the action of the second fluorine atom, namely an F ··· H interaction at the C12 position of DNA. In addition, the series of complexes were tested to evaluate their cytotoxic activities against HeLa, SKOV-3 and L-02 cell lines. Low cytotoxic activities of the complexes were observed before irradiation. However, when irradiation was applied, their cytotoxic activities increased, apparently due to increased cell uptake enhanced by the effect of light, thus positioning these complexes as potential PACT agents.

Noteworthy is the fact that the RU18 complex presented less photocytotoxicity and cell uptake than Ru17 and Ru19 towards the evaluated cell lines, for reasons that are not yet clear.

## 9. Tin Metallodrugs

Gielen et al. were apparently the first to synthesize Sn-based metallodrugs containing fluorinated ligands with anticancer activity. They synthesized various tin complexes by reacting di-n-butyltin oxide in the presence of the mono, di, tri, tetra, and pentafluorinated benzoic acids [82]. The antitumor properties of these complexes were evaluated against the MCF-7 and WiDr cell lines. The ID_50_ values revealed the complexes with the difluorinated ligands to have the best antitumor activity compared with the monofluorinated ones when they were exposed to the MCF-7 cell line. The compound containing the 2,3,6-trifluorobenzoate ligand exhibited activity similar to the aforementioned compounds; however, the tetra and pentafluorinated acids showed lower activity. Against WiDr, all the complexes presented similar activity except the 2,3-difluoro derivative, which was slightly more active.

**Scheme 4:**
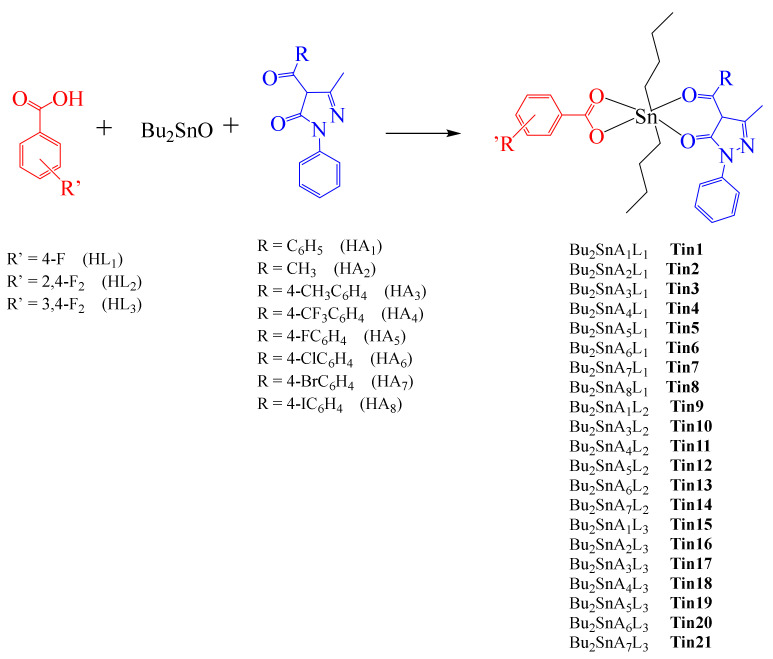
Sn(IV) Tin01–Tin21 fluorinated complexes containing acylpyrazolone and fluorinated benzoate ligands.

On the other hand, Zhao et al. prepared a series of dibutyltin(IV) complexes that combined acylpyrazolone and fluorinated benzoate ligands (Scheme 4) [83].This work was inspired by Ti(IV) species that have exhibited promising in vitro antitumor activities [84] and by previous work reported by Gielen using fluorinated derivatives of benzoic acid (vide supra) [82].

The cytotoxic effect of these complexes was evaluated against the human tumor cell lines KB and HeLa, using Cisplatin as a positive control. All complexes showed better cytotoxicities compared with Cisplatin, with IC_50_ values ranging from 0.05 to 0.54 μM. HeLa cells apparently were more sensitive to the treatment with all complexes. Specifically, Tin2 complex was used as a model to study its apoptotic activity. Hence, using flow cytometry, results showed that complex Tin2 induces apoptosis at concentrations around 1.0 μM.

## 10. Miscellaneous

**Figure 20:**
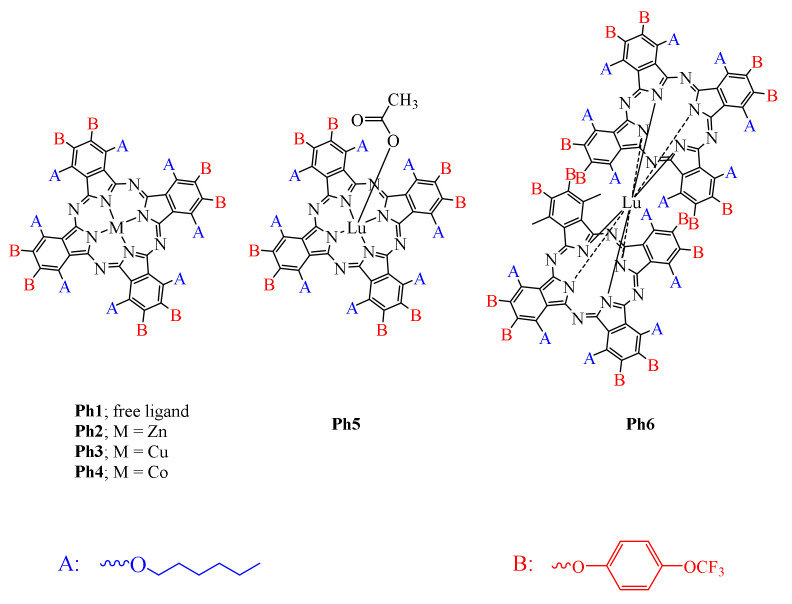
Fluorinated phthalocyanines used as photosensitizer in photochemistry and sono-photochemical experiments.

Phthalocyanines are versatile photosensitizers that have recently received great attention mainly in PDT (photodynamic therapy) for the treatment of cancer [85]. PDT is usually a low-toxic method for healthy cells, minimally invasive and accurate. The interaction of the light-activated photosensitizer with molecular oxygen produces ROS (singlet oxygen and hydroxyl radicals). Although low concentrations of photosensitizer are required, the use of PDT may be limited in deeper lesions, as light cannot penetrate to activate the photosensitizer. In this case, in deeper lesions, the photosensitizer can be activated by ultrasonication (sonophotodynamic therapy: SDT); however, higher concentrations are required, which can be toxic to living cells. Faced with the dilemma of circumventing these drawbacks, both methods (PDT and SDT) have been combined, creating a more efficient treatment for deeper lesions. In this way, Farajzadeh et al. reported a series of fluorinated phthalocyanines containing various metals that were tested as photosensitizing agents in the combination of photodynamic/sonophotodynamic therapies, Figure 20 [85].

Preliminary photophysical and photochemical/sonochemical studies of the compounds showed that those containing closed shell diamagnetic heavy metal ions had a high triplet quantum yield and long triplet lifetime. Compounds containing paramagnetic metals had drawbacks in this regard. For this reason, the photochemistry and sono-photochemical studies were only performed with compounds Ph1, Ph2, and Ph5, using 1,3-diphenylisobenzofuran (DPBF) as singlet oxygen quencher. In general, the samples were irradiated every 5 s with ultrasound (at a frequency of 35 kHz) and/or light (intensity of 7.05 × 1015 photons per s per cm^2^). At the end of the experiment, the absorbance of the quencher was read at 416 nm. The ability of a photosensitizer to produce singlet oxygen is determined by calculating the quantum yield of singlet oxygen (Φ_Δ_). Compound Ph2 (0.45) showed the highest Φ_Δ_ in the photochemical studies (Ph1: 0.39 and Ph5: 0.17). The results obtained in the sono-photochemical experiments (combining both), where the samples were irradiated with ultrasound for 5 s and immediately by light for 5 s, gave the following values of Φ_Δ_ (Ph1: 0.77; Ph2: 0.91, and Ph3: 0.39). It was directly observed that the combined method increased the production of singlet oxygen at least twofold. The determination of the photodegradation quantum yield (Φ_d_) value indicated how stable the photosensitizer was during the irradiation processes (photo and sonochemistry). From the values of Φ_d_, it was determined that the compounds (Ph1: 5.14; Ph2: 4.60, and Ph3: 1.02) were stable to photo- and sono-photochemical degradation. The antioxidant capacity of the compounds was determined by evaluating DPPH radical scavenging. In this experiment, all compounds (Ph1–Ph6) were evaluated. The DPPH radical scavenging activity was evaluated at different concentrations (12.5, 25, 50, 100, and 200 mg/L). In general, each time the concentration was increased, the %DPPH scavenging ability in all the evaluated compounds increased. However, there were interesting findings. In the experiments at concentrations of 50 mg/L, compounds Ph4–Ph6 showed a %DPPH scavenging ability of 100%. Interestingly, when the concentration was increased from 25 to 100 mg/L, increases in the %DPPH scavenging ability values were also observed: for instance, from 78.9 to 100% for compound Ph1, from 83.6 to 100% for compound Ph2, from 76.5 to 100% for compound Ph3, from 90.53 to 100% for compound Ph4, from 99.5 to 100% for compound Ph5, and from 92.6 to 100% for compound Ph6. All compounds showed a %DPPH scavenging ability of 100% at 200 mg/L. The authors compared the scavenging activity of their compounds with similarly reported ones and stated that the series of compounds Ph1–Ph6 showed substantially higher activities. In addition, the DNA cleavage propensity of all compounds was evaluated by gel electrophoresis using plasmid pBR322 DNA in Tris-HCl buffer. The compounds Ph3, Ph5, and Ph6 exhibited single-stranded DNA cleavage capabilities. However, compounds Ph1, Ph2, and Ph4 showed double-strand DNA cleavage faculties. The authors also carried out other types of experiments to assess antimicrobial activity, biofilm inhibition activity, microbial cell viability, and antimicrobial photodynamic activity; however, they will not be discussed here as they fall outside the scope of this review. In general, it can be said that based on the values of Φ_Δ_ (Ph1: 0.77; Ph2: 0.91 and Ph3: 0.39) obtained in the sono-photochemical experiments, they were double, which indicates greater production of singlet oxygen compared with photochemical assays.

**Scheme 5:**
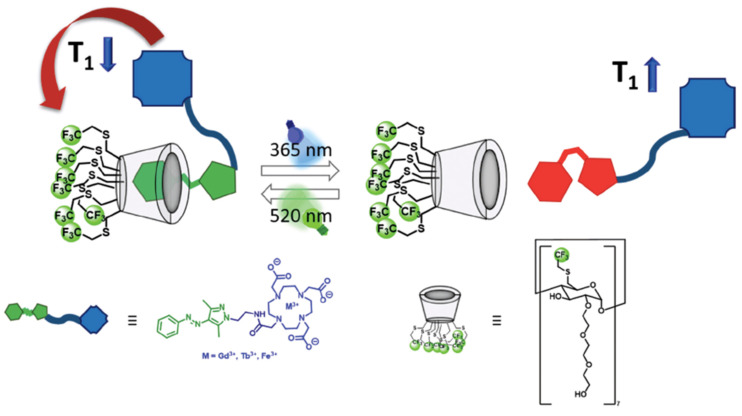
Representation of the photoresponsive modulation of T_1_/T*_2_ relaxation time of FCD and AAP-functionalized DOTA metal complexes by host–guest interaction. Reproduced with permission from [86], RSC, 2021.

On the other hand, one of the drawbacks in using MRI of the ^19^F nucleus is the limitation of not having enough imaging agents. Although the MRI of this nucleus shows no ambiguity caused by the high-water environment, the opposite is true when using MRI with the ^1^H nucleus; few imaging agents have been developed. Agents based on perfluorocarbons (PFC) are good candidates; however, they present solubility problems. When fluorinated agents present solubility problems, emulsions are prepared; however, it must be considered that they can interfere in the experiments. Lipid micro/nanoparticle (NP) formulations can also be used (Section 5, gold metallodrugs). The use of Gd as a ^19^F MRI contrast agent due to its paramagnetic properties has received much attention lately. Analogously, the use of other metals (Ni, Co, and Fe) has also been widely investigated in this regard. In addition, the approach using non-covalent devices to obtain light-sensitive contrast agents has been explored. Cyclodextrins (CDs) are excellent candidates because they can accommodate hydrophobic guest molecules, have high water solubility, and can be easily functionalized. In fact, CDs decorated with multiple Gd ions provoke a short relaxation time. In this sense, the authors reported the synthesis of fluorinated CD (FCD), which can modulate the relaxation time of ^19^F, longitudinal (T_1_) and transversal (T*_2_) by the light-responsive host–guest complexation with arylazopyrazole (AAP)-functionalized 1,4,7,10-tetraazacyclodo-decane-1,4,7,10-tetraacetic acid (DOTA) metal complexes (Scheme 5) [86].

**Figure 21:**
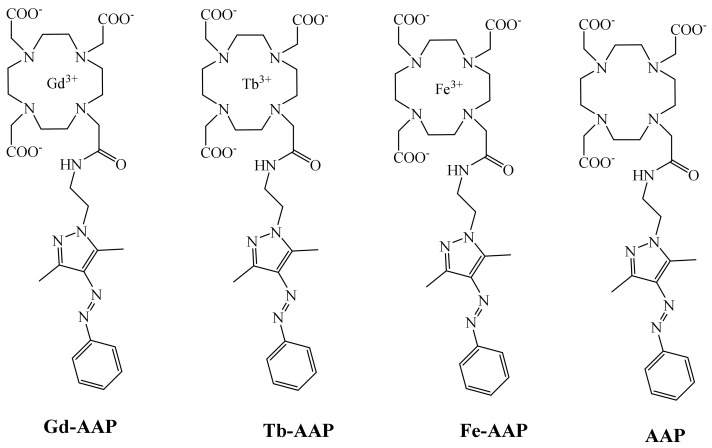
DOTA metal complexes Gd-AAP, Tb-AAP and Fe-AAP.

FCD is a highly soluble compound that contains 21 equivalent fluorine atoms, making it a suitable contrast agent for MRI. The functionalized moiety (APP) works as molecular light-responsive switch with promising photochemical and -physical properties. With the formation of the 1:1 host–guest adduct (non-covalent interactions), it is expected that relaxation times will be decreased by shortening the distance between paramagnetic metal ions and ^19^F in FCD. The E-Z isomerization of the AAP moiety after irradiation at 365 nm will cause dissociation of the adduct due to the formation of the Z isomer, increasing relaxation time. Irradiation at 520 nm reverses the process. Firstly, the relaxation time (T_1_/T*_2_) of FCD was evaluated to observe its properties as a contrasting agent. FDC (T_1_ = 270 ms) exhibited shorter relaxation times compared to other ^19^F contrast agents such as perfluoro-15-crown-ether (T_1_ = 1069 ms). This makes FCD a good candidate. Determination of the transversal relaxation time of FCD showed a value of T*_2_ = 3.00 ms, which is indeed a short time, but not as short as expected. Micelle formation is thought to impede this. The design of metal complexes based on DOTA Gd-AAP, Tb-AAP, and Fe-AAP makes them suitable guests for FCD (Figure 21). These metals were selected because all three have recently shown interesting properties as MRI contrast agents, either because they are suitable for reducing the relaxation time of close-by nuclei, present a strong luminescence and a large magnetic moment, or have low toxicity. Electron paramagnetic resonance (EPR) studies showed that Fe-AAP is found as intermediate-spin Fe^3+^. Binding constants (host–guest adducts) were determined by isothermal titration calorimetry: K_a_ = 9.90 × 10^2^ M^−1^ for Gd-AAP, K_a_ = 1.81 × 10^2^ M^−1^ for Tb-AAP, and K_a_ = 7.53 × 10^2^ M^−1^ for Fe-AAP.

Light-sensitive host–guest experiments on the isomerization of AAP fragments in both ways (Scheme 5) at 365 nm and 520 nm of UV light showed interesting results. Different percentages of reisomerization to the E-isomer were observed depending on the compound: while Gd-AAP and Tb-AAP regain around 90% of their initial intensity, Fe-AAP only recovers 74%. Carrying out these photoisomerization experiments for up to three cycles, Gd-AAP and Tb-AAP were shown to be fully reversible; however, Fe-AAP showed problems in this sense in each cycle of isomerization. In addition, thermal stability was evaluated on the basis of half-life. Gd-AAP exhibited the highest half-life (τ_1/2_ = 4 d) and the other two compounds (Tb-AAP and Fe-AAP) showed a decrease in these values (τ_1/2_ = 29 h and τ_1/2_ = 7 h). Due to the paramagnetic nature of the three metals in Gd-, Tb- and Fe-AAP, it was impossible to analyze the photostationary state (PSS). However, for these purposes, PSS was analyzed in the unmetalled compound APP (Figure 21). Quantitative photo-switching in both directions (about 98%) was estimated by ^1^H NMR experiments. Comparing these results with those obtained by the UV experiments indicated that the photophysical properties will be affected by the complexation of metal ions. According to all of these results, Gd-APP and Tb-APP showed the most outstanding photophysical properties. In the case of Fe-APP, it was observed that the photoisomerization processes were carried out incompletely. Determinations of (T_1_/T*_2_) relaxation time to the host-guest adducts of the FCD were carried out by means of NMR studies (inversion-recovery). Based on ^19^F-FDC signal tracking, after the addition of Gd-APP or Fe-APP, there was a dramatic decrease in T_1_. A decrease of 44% (T_1_ =151 ms) for Gd-APP, and of 59% for Fe-APP, was observed (T_1_ = 111 ms). In the case of Tb-APP, a minimal decrease in T_1_ was observed (T_1_ = 244 ms). The addition of only the Gd-DOTA moiety as a negative control had no effect on the relaxation time for FCD (T_1_ = 264 ms; T*_2_ = 2.90 ms). This last experiment indicated the strong effect that the non-covalent host–guest formation of FCD with the AAP-functionalized DOTA metal complex Gd-AAP has on the relaxation time since it approximates the paramagnetic metals with the ^19^F nuclei. Specifically, it was observed that the formation of this host–guest complex is concentration-dependent. At low concentrations, it was determined that the host-guest adduct is not preferentially formed, resulting in a higher relaxation time for the FCD. Light-responsiveness investigations of the formation of host–guest complexes were carried out at 365 nm (Scheme 5). After being irradiated, the T_1_ values increased: 130% for Fe-APP (256 ms) and 51% for Gd-APP (228 ms). The increase in T_1_ relaxation time results from the dissociation of the host–guest complex, due to complete conversion to the Z-isomer. In the formation of the host-guest adduct with Tb-APP, there were no substantial changes in T_1_ values. Irradiation at 560 nm reversed the process (Figure 21) towards the formation of the E-isomer. Gd-AAP (T_1_ = 155 ns) did not present a problem to completely carry out the conversion of the Z isomer to E, the opposite of the case with Fe-AAP, in which the process was not fully carried out; therefore, a less-pronounced decrease in T_1_ relaxation time was observed. The determination of T*_2_ was evaluated by means of ^19^F NMR; Gd-AAP (T*_2_ = 0.642 ms) presented the greatest change in this value, followed by Tb-AAP (T*_2_ = 1.81 ms) and finally Fe-AAP (T*_2_ = 2.26 ms). The authors indicated that there is a large effect on relaxation time T*_2_ due to the influence of metal ions, and this is consistent with similar systems previously reported. They also pointed out that both T_1_ and T*_2_ can be controlled by irradiation and that such supramolecular systems may create a precedent in the development of new ^19^F MRI contrast agents.

## 11. Conclusions

In conclusion, this review described an approach-denominated structural optimization of the ligand, with special emphasis on ligand fluorination. These methods often allow radical changes in various physicochemical and biological properties of the metal complexes, conferred by these fluorinated ligands, although the mere introduction of fluorine atoms on the ligands does not always guarantee the desired changes in properties, as was the case with the series of complexes Pt12–Pt14 (Scheme 3), where a fluorine atom was introduced axially in the hope of changing the lipophilicity of these species but without success, although this is regularly achieved when the ligand has carbon–fluorine bonds. Other beneficial changes caused by the introduction of fluorine involve the analysis and potential monitoring of these new complexes by several methods such as ^19^F NMR, ^19^F MRI or ^18^F positron emission tomography, which are extremely useful in medicine. This was exemplified in this review by the gold NPs described in Section 3, which can be detected by ^19^F MRI.

The introduction of fluorine on the ligands, and thus, on the structure of metal complexes represents a rational, relevant approach to the potential enhancement and modulation of the properties of metallodrugs, namely their solubility and lipophilicity. However, this approach must be complemented with the aid of molecular docking among other techniques to facilitate the proper design of these functional compounds.

## Data Availability

No new data were created or analyzed in this study. Data sharing is not applicable to this article.

## Abbreviations

CF_3_-dppz	11- (trifluoromethyl)-dipyrido [3,2-a:2′,3′-c] phenazine
CDs	cyclodextrins
CLSM	confocal laser scanning microscopy
Cco	cytochrome oxidase
CRT	calcium storage protein calreticulin
DAMPs	damage-associated molecular pattern signals
dmba	dimethylaminomethyl-phenyl
DNA	deoxyribonucleic acid
DFT	density functional theory
DPBF	1,3-diphenylisobenzofuran
F-diCO	1,3-dicarbonyl
dfppy	2-(2,4-difluorophenyl)pyridine
dppbz	1,2-bis(diphenylphosphino)benzene
dppz	dipyrido[3,2-a:2′,3′-c]phenazine
dppe	diphenylphosphino)ethane
dppm	diphenylphosphino)methane
DPPC	1,2-dipalmitoyl-sn-glycero-3-phosphocholine
EB	ethidium bromide
EC_50_	half-maximal effective concentration
E_1/2_	half-wave potentials
EG	ethylene glycol
Φ_P_	quantum yield
3F-BnNH_2_	3-fluorobenzylamine
FCD	fluorinated cyclodextrin
F-dppz	11-fluoro-dipyrido [3,2-a:2′,3′-c] phenazine
F2-dppz	11,12-difluorodipyrido [3,2-a:2′,3′-c] phenazine
F-enm	fluorinated enaminone
3F-salen	3-fluorosalicylaldehyde ethylenediamine
HCC	hepatocellular carcinoma
IC_50_	half-maximal inhibitory concentration
ICD	immunogenic cell death
JNK	c-Jun NH2-terminal kinase
K_app_	apparent binding constants
K_b_	intrinsic binding constants
K_q_	quenching constant
KP1019	indazolium *trans*-[tetrachlorobis(1*H*-indazole)ruthenate(III)]
MeIm	N-methylimidazole
MRI	magnetic resonance imaging
MTT	thiazolyl blue tetrazolium bromide
NMR	Nuclear magnetic resonance
NAMI-A	*trans*-[tetrachloro(DMSO)(imidazole)ruthenate(III)]
NHC	N-heterocyclic carbenes
NKP-1339	sodium salt analogue of KP1019
NP	nanoparticle
OLED	organic light-emitting devices
PACT	photoactivated chemotherapy
PDT	photodynamic therapy
PFC	perfluorocarbons
phen	1,10-phenanthroline
PI	propidium iodide
pic-TPA	triphenylamine tethered pyridine-2-carboxylate
PPS	photostationary state
QDs	quantum dots
RAPTA	ruthenium (II)-arene complexes (pta= 1,3,5-triaza-7-phosphaadamantane)
RF	resistant factor
ROS	reactive oxygen species
SCXRD	single crystal X ray diffraction
SDT	sonophotodynamic therapy
SER	sensitization enhancement ratio
SOD	Zn, Cu-super-oxide dismutase
tfpa	3,3,3-trifluoropropanoate
tfacen	trifluoroacetylacetone ethylenediamine
TFPPy	2-(3,4,5-trifluorophenyl) pyridine
TFPQ	2-(3,4,5-trifluorophenyl) quinoline
Φ_Δ_	quantum yield of singlet oxygen
